# Genomic and phenotypic characterization of 26 novel marine bacterial strains with relevant biogeochemical roles and widespread presence across the global ocean

**DOI:** 10.3389/fmicb.2024.1407904

**Published:** 2024-05-28

**Authors:** Xavier Rey-Velasco, Teresa Lucena, Ana Belda, Josep M. Gasol, Olga Sánchez, David R. Arahal, María J. Pujalte

**Affiliations:** ^1^Institut de Ciències del Mar (ICM-CSIC), Barcelona, Catalunya, Spain; ^2^Departamento de Microbiología y Ecología, Universitat de València, València, Spain; ^3^Departament de Genètica i Microbiologia, Facultat de Biociències, Universitat Autònoma de Barcelona, Bellaterra, Spain

**Keywords:** taxonomy, genomics, phylogenomics, isolates, marine bacteria, biogeochemistry, biogeography

## Abstract

Prokaryotes dominate global oceans and shape biogeochemical cycles, yet most taxa remain uncultured and uncharacterized as of today. Here we present the characterization of 26 novel marine bacterial strains from a large isolate collection obtained from Blanes Bay (NW Mediterranean) microcosm experiments made in the four seasons. Morphological, cultural, biochemical, physiological, nutritional, genomic, and phylogenomic analyses were used to characterize and phylogenetically place the novel isolates. The strains represent 23 novel bacterial species and six novel genera: three novel species pertaining to class *Alphaproteobacteria* (families *Rhodobacteraceae* and *Sphingomonadaceae*), six novel species and three new genera from class *Gammaproteobacteria* (families *Algiphilaceae*, *Salinispheraceae*, and *Alteromonadaceae*), 13 novel species and three novel genera from class *Bacteroidia* (family *Flavobacteriaceae*), and one new species from class *Rhodothermia* (family *Rubricoccaceae*). The bacteria described here have potentially relevant roles in the cycles of carbon (e.g., carbon fixation or energy production via proteorhodopsin), nitrogen (e.g., denitrification or use of urea), sulfur (oxidation of sulfur compounds), phosphorus (acquisition and use of different forms of phosphorus and remodeling of membrane phospholipids), and hydrogen (oxidation of hydrogen to obtain energy). We mapped the genomes of the presented strains to the Tara Oceans metagenomes to reveal that these strains were globally distributed, with those of the family *Flavobacteriaceae* being the most widespread and abundant, while *Rhodothermia* being the rarest and most localized. While molecular-only approaches are also important, our study stresses the importance of culturing as a powerful tool to further understand the functioning of marine bacterial communities.

## 1 Introduction

Prokaryotes are the most abundant cells in the ocean (*ca.* ~10^29^; Whitman et al., 1998), and bacteria have been estimated to represent around ~22% of the ocean’s biomass (Bar-On and Milo, 2019). They possess a wide metabolic diversity, which makes them drive the ocean’s biogeochemical cycles (Falkowski et al., 2008). In fact, prokaryotes are likely responsible for most of the ocean’s respiration (Del Giorgio and Duarte, 2002) and have a major role in key steps of the cycles of carbon, nitrogen, sulfur, phosphorus, hydrogen, iron, and other metals (Gasol and Kirchman, 2018). Microbes are largely responsible for the transformation of inorganic carbon into organic matter via primary production and the transformation of this production into particulate organic matter, supplying carbon and nutrients upwards through the food web (Azam et al., 1983; Farooq, 1998; Fenchel, 2008).

While it has been estimated that there are 10^12^ microbial species on Earth (Locey and Lennon, 2016), only 22,525 prokaryotic species have been validly published to date (List of Prokaryotic Names with Standing Nomenclature, 11 January 2024), in part because most environmental (including marine) bacteria remain uncultured (Martiny, 2019; Steen et al., 2019).

Isolation is not only a prerequisite for the valid publication of novel taxa (Parker et al., 2019), but it is also an essential tool that allows understanding of the ecology of marine bacteria by providing the possibility to perform physiological experiments to test ecological hypotheses, unveiling their biogeochemical potential by providing access to their whole genomes (Giovannoni and Stingl, 2007), and allowing them to answer complex evolutionary questions (Imachi et al., 2020). Thus, more efforts are needed in culturing marine bacteria and comparing their DNA to environmental sequence data, which provides valuable information about the global context and ecology of the cultured taxa. Moreover, this task has become easier in the last few years with the massive amount of data generated in global sampling expeditions such as Tara Oceans (Pesant et al., 2015) or Malaspina (Duarte, 2015).

We have recently obtained a large culture collection from Blanes Bay (NW Mediterranean) seawater microcosm experiments run in the four astronomical seasons in which we tested the impact of the reduction in grazing and viral pressure, the increase in nutrient availability, and the presence/absence of light on the culturability of marine bacteria (Rey-Velasco et al., 2023). In this culturing effort, we could isolate taxa that represented the top-abundant fractions of the population after some of the manipulations, and we isolated a set of bacteria with less than 97% identity in their 16S rRNA gene sequences to cultured taxa in public databases. Based on that work, we present a comprehensive description of 26 novel marine bacterial strains, including their phenotypic, genomic, and phylogenomic characterization, their potential relevance in biogeochemical cycles, and their distribution across global oceans.

## 2 Materials and methods

### 2.1 Isolation of the strains

All strains were isolated from surface seawater samples taken in February, April, July, and November 2017 from the Blanes Bay Microbial Observatory (BBMO) in the NW Mediterranean (41°40′N, 2°48′E), approximately 1 km offshore. The seawater samples were subjected to experimental manipulations to reduce predators and viruses and increase nutrient availability, then incubated for 36–48 h as described in Sánchez et al. (2020). Subsamples of 1 mL were stored in cryovials at −80°C with 75 μL DMSO until the time of isolation. The isolation was carried out between November 2020 and January 2022 using samples from the initial and final times of the experiments, as reported in Rey-Velasco et al. (2023). Briefly, aliquots of 100 μL of undiluted, 1:10 and 1:100 diluted seawater were spread in triplicate on Marine Agar 2216 (MA; Difco) and Marine Reasoner’s 2A Agar (mR2A) plates and incubated at room temperature (20–25°C) until no more colonies appeared (maximum 30 days). Colonies with different morphologies were selected, streaked on new agar plates, transferred to liquid cultures, and stored with 25% glycerol in cryovials at −80°C.

### 2.2 Identification and selection of the strains

The amplification and sequencing of the 16S rRNA gene were carried out as described in Rey-Velasco et al. (2023). Briefly, the DNA of each isolate was extracted from 100 μL of liquid culture by thermal shock and was used for PCR amplification of the nearly complete 16S rRNA gene with primers 27Fmod (5′-AGR GTT TGA TCM TGG CTC AG-3′) and 1492Rmod (5′-TAC GGY TAC CTT GTT AYG ACT T-3′) from Page et al. (2005). When faint or no bands were observed on agarose gel electrophoresis following PCR, DNA extraction was repeated with the DNeasy Blood & Tissue Kit (Qiagen) following the manufacturer’s recommendations. Purification and OneShot Sanger sequencing of PCR products were carried out by Genoscreen (Lille, France) with the two above-mentioned primers. A total of 1,643 isolates were sequenced in the original study.

These 16S rRNA gene sequences were manually quality-checked, trimmed, and assembled with Geneious software v. 2022.0.1 (Kearse et al., 2012). An initial classification of the near-complete 16S rRNA gene sequences was made with the SINA aligner (Pruesse et al., 2012) against the SILVA database (release 138.1). Isolate sequences were also submitted to BLASTn v. 2.12.0+ (Altschul et al., 1990) against a subset of the RDP database (release 11; Cole et al., 2014) containing only cultured taxa, and another one containing only uncultured taxa, as shown in Figure 1 of Rey-Velasco et al. (2023). The sequences that presented less than 97% identity to cultured taxa according to these data were assessed against type strains using the Identifier tool of EzBioCloud (Yoon et al., 2017). Considering this threshold, we selected for the analyses described here 26 isolates that had less than 96.6% similarity with any type of strain.

### 2.3 Whole-genome sequencing, assembly, and characterization

The genomic DNA of the 26 selected strains was extracted using the Bacterial DNA Preparation Kit (Jena Bioscience), and its integrity and concentration were checked using DNA gel electrophoresis and a Qubit 1 Fluorometer (Invitrogen), respectively. Further quality control, library preparation, and whole-genome sequencing were carried out using different methodologies: three of the strains were sequenced in the Central Service of Support to Experimental Research (SCSIE) of the University of Valencia (Valencia, Spain): strains S334^T^ and S365 with Illumina MiSeq (2× 250 bp reads) aiming to a coverage of 120× and strain S356^T^ with Sequel PacBio RS II technology (SMRT Link version 7.0) aiming to a coverage of 500×. The other 23 isolates were sequenced in the Center Nacional d’Anàlisi Genòmica (CNAG) with an Illumina MiSeq sequencer (2× 300 bp, Illumina) aiming for a coverage of 120 ×.

The genome of strain S356^T^ was assembled with the Hierarchical Genome Assembly Process (HGAP4) *de novo* assembly analysis application. The genomes of strains S334^T^ and S365 were assembled as in (Macián et al., 2021). To assemble the other 23 genomes, we used the Nextflow pipeline bacass v.2.0.0 (Peltzer et al., 2021) from the nf-core framework (Ewels et al., 2020). The pipeline runs an automated workflow that uses Skewer (Jiang et al., 2014) to quality trim the reads, performs basic sequencing QC using FastQC,^1^ and assembles the reads with Unicycler (Wick et al., 2017). Assembly contamination was checked with Kraken2 (Wood et al., 2019), and its quality was assessed using QUAST (Gurevich et al., 2013).

All genomes were quality assessed with CheckM v.1.2.2 (Parks et al., 2015), which also provided basic genomic data such as %GC, genome length, or number of predicted genes. Genomes were annotated using Prokka v.1.14.6 (Seemann, 2014) and then functionally annotated with eggNOG-mapper v.2.1.10 (Cantalapiedra et al., 2021) based on eggNOG orthology data (Huerta-Cepas et al., 2019) and DIAMOND sequence searches (Buchfink et al., 2021). Taxonomic classification was obtained using GTDB-Tk v.2.3.2 (Chaumeil et al., 2020, 2022), relying on release 214 of the GTDB database (Parks et al., 2022). All these pipelines and CheckM were run using custom makefiles available on GitHub.^2^

### 2.4 16S rRNA gene sequence analysis

Sequences of 16S rRNA genes were extracted using seqkit v.2.1.0 (Shen et al., 2016) and compared with the sequences initially obtained by Sanger sequencing in order to assure the authenticity of the genomes. Their completion was tested with the Identifier tool of EzBioCloud (Yoon et al., 2017). In the case of strain W364, a part of the 16S rRNA gene sequence obtained by Sanger sequencing was added to the one extracted from the genome in order to increase its completion, after checking their overlap with BLASTn v. 2.12.0+. In the case of strain P117^T^, the 16S rRNA gene sequence extracted from the genome came in two pieces, which represented two exact halves of the gene according to their alignment with the sequence of the same gene obtained by Sanger sequencing; therefore, they were joined together to form a 100% complete sequence. The final set of 16S rRNA gene sequences was taxonomically classified with GTDB database rs214 (Parks et al., 2022) using the Nextflow pipeline ampliseq v.2.7.1 (Straub et al., 2023) from the nf-core framework (Ewels et al., 2020).

### 2.5 Phenotypic characterization

Colony morphology and pigmentation were recorded after 24 h, 1 week MA cultures, depending on the growth rate of each strain. Cell morphology and motility were determined by optical microscopy in wet mounts prepared from MA cultures of the same age. In some cases, cell morphology was confirmed by basic fuchsin staining. Gram determinations were made by lysis tests in 3% KOH. For the *Flavobacteriaceae* strains, flexirubin-type pigmentation was tested with 20% KOH according to the method of Bernadet et al. (2002).

Growth in MA and mR2A was determined by incubating the plates at 26°C in the dark. The temperature ranges for growth (4, 15, 28, 37, and 40°C) were determined in MA incubated for up to 2 days at 40°C and 3 weeks in the rest. Ranges of salinity (0, 0.5, 1, 1.5, 2, 3, 4, 5, 6, 7, 8, 9, 10, 11, 12%) and growth in 2% (w/v) NaCl as sole salt were determined after incubation for up to 2 weeks, first in a medium composed of 15 g/L agar, 5 g/L peptone, and 1 g/L yeast extract supplemented with the appropriate Sea Salts (Sigma) or NaCl quantity and, for some of the strains that did not grow well in this formulation, in Marine Agar supplemented with NaCl to reach the target concentration. Extracellular hydrolytic activities on casein, starch, Tween-80, and DNA were determined as in Pujalte et al. (2018). Extracellular hydrolysis of alginate was determined in MA supplemented with 2 g/L sodium alginate. Sole carbon and energy sources used for growth were tested on simplified Basal Medium Agar (Baumann and Baumann, 1981) composed of 20 g/L Sea Salts (Sigma), 1 g/L NH_4_Cl, 75 mg/L K_2_HPO_4_, 30 mg/L FeSO_4_·7H_2_O, 0.1 g/L yeast extract, 12 g Purified Agar, and 2 g/L carbon source. The medium used to test carbon sources in strains S334^T^, S356^T^, and S365 did not contain yeast extract. The oxidase test was performed with Oxoid Oxidase Strips, and catalase was tested with 10 vol. H_2_O_2_. API 20NE (for all strains except S356) and API ZYM strips (only 10 strains due to the product discontinuation) were inoculated as in Pujalte et al. (2018). Main polar lipids and respiratory quinones were determined by manually searching their synthesis enzymes in their Prokka genome annotation files and on the RAST server (Aziz et al., 2008). Other relevant taxogenomic characteristics were searched using the same methods.

### 2.6 Inference of phylogeny

We used the Type Strain Genome Server (TYGS; Meier-Kolthoff and Göker, 2019) to compute digital DNA–DNA hybridization (dDDH) values between the novel strains and related type strains, then selected the 25 closest type strains for each isolate and grouped them according to their classification into classes. The accession numbers of these reference strains were obtained from the TYGS output, and their genome sequences were downloaded using NCBI Datasets.^3^ Average nucleotide identity (ANI) was computed with fastANI (Jain et al., 2018), and average aminoacid identity (AAI) was calculated with EzAAI (Kim D, et al., 2021) between all the strains, the novel and the reference ones, within each group. The Kostas Lab ANI/AAI matrix tool (Rodriguez-R and Konstantinidis, 2016) was used to cross-check some of the AAI values. Phylogenomic trees were computed for each group with UBCG2 (Kim J, et al., 2021), including outgroups and, in some cases, relevant type strains that were missing in the original TYGS search. The trees were graphically represented and customized using the Interactive Tree of Life (iTOL; Letunic and Bork, 2021). Nodes with gene support values lower than 60 (out of 81 core gene trees) were deleted.

### 2.7 Screening of biogeochemically relevant genes and geographic distribution

We generated a curated list of biogeochemically relevant genes involved in the metabolism of carbon, nitrogen, phosphorus, sulfur, iron, and hydrogen based on previous findings (e.g., Ferrera et al., 2015; Satinsky et al., 2017; Auladell et al., 2023). This list is available in Supplementary Table S1. We searched the KEGG orthologs (KO; Jin et al., 2023), Pfams (Mistry et al., 2021), or Clusters of Orthologous Genes (COGs; Tatusov et al., 2000) that these genes represented in eggNOG-mapper annotations.

The geographic distribution of the strains was computed by calculating the coverages of the novel genomes in Tara Oceans metagenomes (Pesant et al., 2015) with CoverM v.0.6.1^4^ using BWA-MEM v.0.7.17-r1188 (Li, 2013) as the mapping algorithm. Mapping with BWA failed for a few files because they had unmatching read names; therefore, they had to be repaired with BBMap v.38.90 (Bushnell, n.d.) and mapped again. We set 25% breadth of coverage as a threshold to decide whether a strain was present in a given sample.

Output tables were sorted and plots were generated in R software v.4.1.3 (Team, R. C, 2022) and RStudio v.1.3.1093 (Team, R. S, 2020), mainly with packages *tidyverse* v.1.3.1 (Wickham et al., 2019), *qdap* v.2.4.3 (Rinker, 2020), *magrittr* v.2.0.3 (Bache and Wickham, 2022), *maps* v.3.4.1.1 (Becker and Wilks, 2023), and *mapdata* v.2.3.1 (Becker and Wilks, 2022).

## 3 Results

### 3.1 Overview of the strains and genome features

The 26 strains characterized here are listed in Table 1, along with their taxonomic affiliation and main genomic features. Fourteen of them pertain to the *Flavobacteriaceae* (class *Bacteroidia*); seven are *Gammaproteobacteria* of families *Alteromonadaceae*, *Salinispheraceae*, and *Algiphilaceae*; three belong to families *Roseobacteraceae*, *Sphingomonadaceae*, and *Erythrobacteraceae* from class *Alphaproteobacteria*; and two affiliate with *Rubrivirga* of the *Rhodothermia* class. Strain S334^T^ has the largest genome (4.57 Mpb), while the smallest (2.84 Mpb) is that of strain W242^T^, both *Flavobacteriaceae*. The highest G + C content (73.7%) was observed in strains F394^T^ and S365, while *Flavobacteriaceae* strains contained the lowest percentages, with strain W332^T^ having the minimum with 32.5%.

**Table 1:** Overview of the strains described in this study, their taxonomy, and main genomic characteristics.

Strain	Class, order, and family	Genome size (Mbp)	DNA G + C (mol%)	Number of genes	Accession
*Tropicimonas omnivorans* F158^T^	*Alphaproteobacteria*; *Rhodobacterales*; *Rhodobacteraceae*	3.94	66.9	3,698	GCA_031846565.1
*Stakelama saccharohpila* W311^T^	*Alphaproteobacteria*; *Sphingomonadales*; *Sphingomonadaceae*	3.19	65.8	2,921	GCA_032229225.1
*Croceicoccus esteveae* F390^T^	*Alphaproteobacteria*; *Sphingomonadales*; *Sphingomonadaceae*	2.86	60.7	2,730	GCA_031846435.1
*Banduia mediterranea* W345^T^	*Gammaproteobacteria*; *Nevskiales*; *Algiphilaceae*	3.84	64	3,634	GCA_031846245.1
*Spectribacter acetivorans* P385^T^	*Gammaproteobacteria*; *Nevskiales*; *Salinisphaeraceae*	3.30	65.7	3,171	GCA_031846305.1
*Sprectribacter hydrogenooxidans* W335^T^	*Gammaproteobacteria*; *Nevskiales*; *Salinisphaeraceae*	3.29	65.6	3,135	GCA_031846275.1
*Glaciecola petra* P117^T^	*Gammaproteobacteria*; *Enterobacterales*; *Alteromonadaceae*	4.05	39.9	3,499	GCA_031846345.1
*Brumicola blandensis* W364	*Gammaproteobacteria*; *Enterobacterales*; *Alteromonadaceae*	3.94	42.6	3,458	GCA_031846205.1
*Brumicola blandensis* W409^T^	*Gammaproteobacteria*; *Enterobacterales*; *Alteromonadaceae*	4.01	42.6	3,515	GCA_031846165.1
*Thalassotalea castellviae* W431^T^	*Gammaproteobacteria*; *Enterobacterales*; *Alteromonadaceae*	3.96	37.2	3,434	GCA_031846185.1
*Patiriisocius hiemis* W242^T^	*Bacteroidia*; *Flavobacteriales*; *Flavobacteriaceae*	2.84	33.7	2,668	GCA_031846325.1
*Urechidicola vernalis* P050^T^	*Bacteroidia*; *Flavobacteriales*; *Flavobacteriaceae*	3.14	33.5	2,790	GCA_031846385.1
*Microcosmobacter mediterraneus* W332^T^	*Bacteroidia*; *Flavobacteriales*; *Flavobacteriaceae*	3.00	32.5	2,724	GCA_031846265.1
*Asprobacillus argos* S356^T^	*Bacteroidia*; *Flavobacteriales*; *Flavobacteriaceae*	3.18	35.2	2,878	GCA_032248395.1
*Autumnicola tepida* F363^T^	*Bacteroidia*; *Flavobacteriales*; *Flavobacteriaceae*	4.37	40	3,973	GCA_031846485.1
*Autumnicola musiva* F117^T^	*Bacteroidia*; *Flavobacteriales*; *Flavobacteriaceae*	4.52	38.3	4,022	GCA_031846585.1
*Autumnicola patrickiae* F188^T^	*Bacteroidia*; *Flavobacteriales*; *Flavobacteriaceae*	4.35	38.4	3,935	GCA_031846525.1
*Autumnicola edwardsiae* F297^T^	*Bacteroidia*; *Flavobacteriales*; *Flavobacteriaceae*	3.83	38.9	3,710	GCA_031846505.1
*Autumnicola lenta* F260^T^	*Bacteroidia*; *Flavobacteriales*; *Flavobacteriaceae*	3.93	38.6	3,522	GCA_031846515.1
*Autumnicola psychrophila* F225^T^	*Bacteroidia*; *Flavobacteriales*; *Flavobacteriaceae*	3.96	38.4	3,479	GCA_031846645.1
*Croceitalea rosinachiae* F388^T^	*Bacteroidia*; *Flavobacteriales*; *Flavobacteriaceae*	3.60	36.4	3,304	GCA_031846445.1
*Croceitalea vernalis* P007^T^	*Bacteroidia*; *Flavobacteriales*; *Flavobacteriaceae*	3.38	34.7	3,041	GCA_031846405.1
*Croceitalea vernalis* P059	*Bacteroidia*; *Flavobacteriales*; *Flavobacteriaceae*	3.29	34.6	2,956	GCA_031846365.1
*Pricia mediterranea* S334^T^	*Bacteroidia*; *Flavobacteriales*; *Flavobacteriaceae*	4.57	47	3,828	GCA_032248455.1
*Rubrivirga litoralis* F394^T^	*Rhodothermia*; *Rhodothermales*; *Rubricoccaceae*	3.72	73.7	3,238	GCA_031846415.1
*Rubrivirga litoralis* S365	*Rhodothermia*; *Rhodothermales*; *Rubricoccaceae*	3.93	73.7	3,432	GCA_032248475.1

Extended genome features and quality values for all the strains are reported in Supplementary Table S2. The genome completeness reported by CheckM was always >96%, with an average of 98.94 ± 0.82%, ranging from 99.89 to 96.72%. Mean contamination was 0.79 ± 0.69% (range 0–3.01%). All the 16S rRNA genes obtained from the genomes matched 100% with those previously obtained by Sanger sequencing, and all but two scored 100% completion in the EzBioCloud identifier. The exceptions were strains W364 and S356^T^ with 95.1 and 98.1% completeness, respectively.

### 3.2 Phylogeny

The full taxonomic classification of the 16S rRNA genes (with EzBioCloud identifier and GTDB) and of the genomes (with GTDB) for each strain is shown in Supplementary Table S3. Pairwise ANI values of strains belonging to the same species, P007^T^–P059, W364–W409^T^, and F394^T^–S365, are >95% (Supplementary Table S4). The highest ANI value was 99.36% between strains W364^T^ and W409. Phylogenomic comparisons with the closest type strains (Supplementary Table S2 for the closest strains; Supplementary Table S5 for all results) showed no dDDH values higher than 70% nor ANI values higher than 95% for any of the tested genomes. The closest AAI value for strain W345^T^ was 62.95% (*Fontimonas thermophila* DSM 23609^T^). In the case of strains W409^T^ and W364, their closest AAI values were, respectively, 76.85 and 76.84%, both to *Glaciecola nitratireducens* FR1064^T^. Strains W335^T^ and P385^T^ had, respectively, *Salinisphaera halophila* YIM 95161^T^ (63.99%) and *Salinisphaera orenii* MKB5^T^ (64.07%) as their closest strains in terms of AAI. Strain S356^T^ presented an AAI of 72.13% with *Polaribacter septentrionalilitoris* ANORD1^T^. The closest AAI for strains F363^T^, F117^T^, F188^T^, F297^T^, F260^T^, and F225^T^ ranged from 74.24 to 75.17% with strains *Salegentibacter mishustinae* KCTC 12263^T^, *Salegentibacter agarivorans* DSM 23515^T^, and *Salegentibacter salarius* DSM 23401^T^.

**Figure 1:**
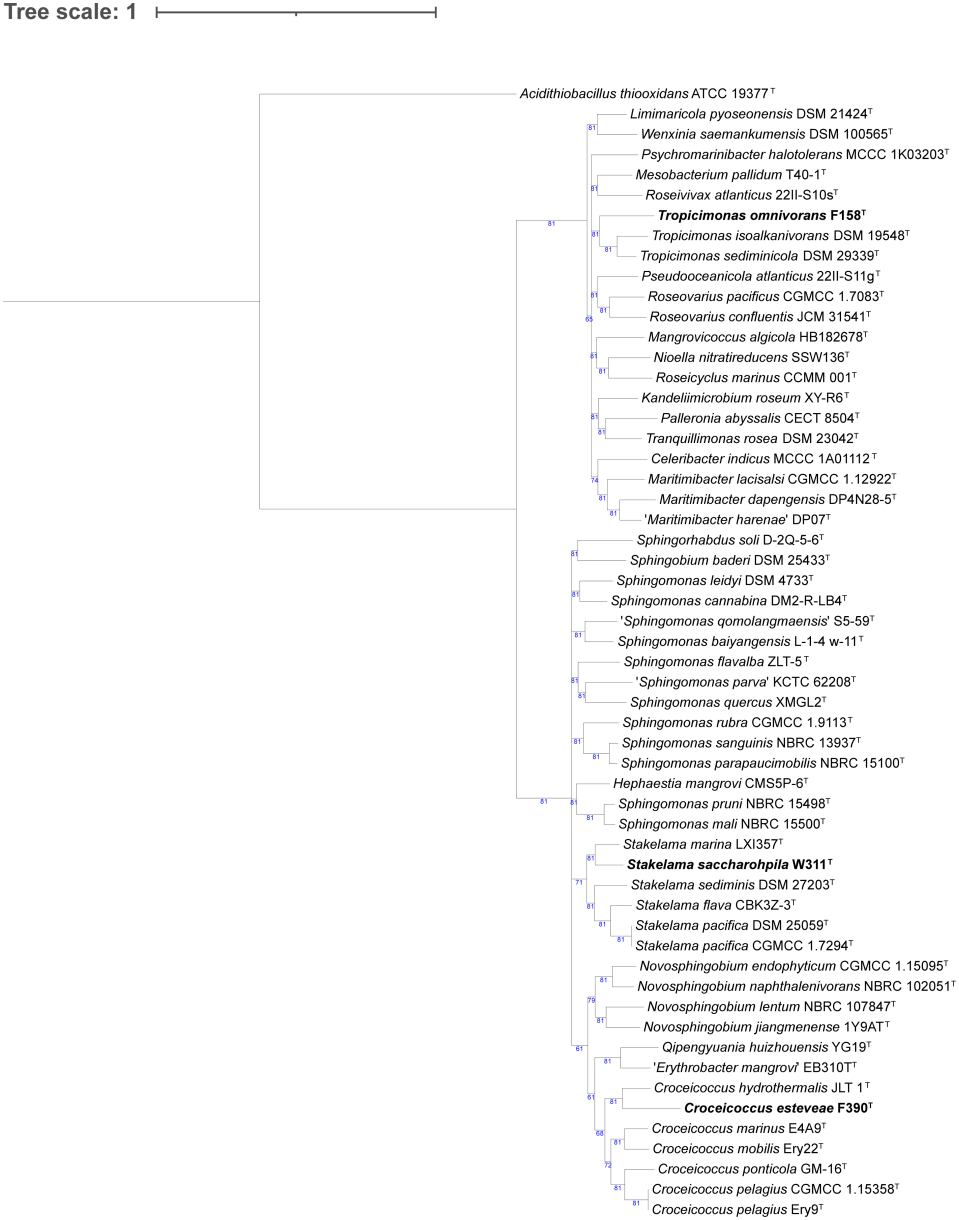
Phylogenomic tree generated with UBCG2 (Kim J, et al., 2021) of the strains belonging to the class *Alphaproteobacteria* and their closest neighbors according to the TYGS (Type Strain Genome Server). The strains characterized in this study are highlighted in bold. Accession numbers for these genomes can be found in Supplementary Table S5. The numbers at the nodes indicate the gene support index, with 81 being the maximum value.

**Figure 2:**
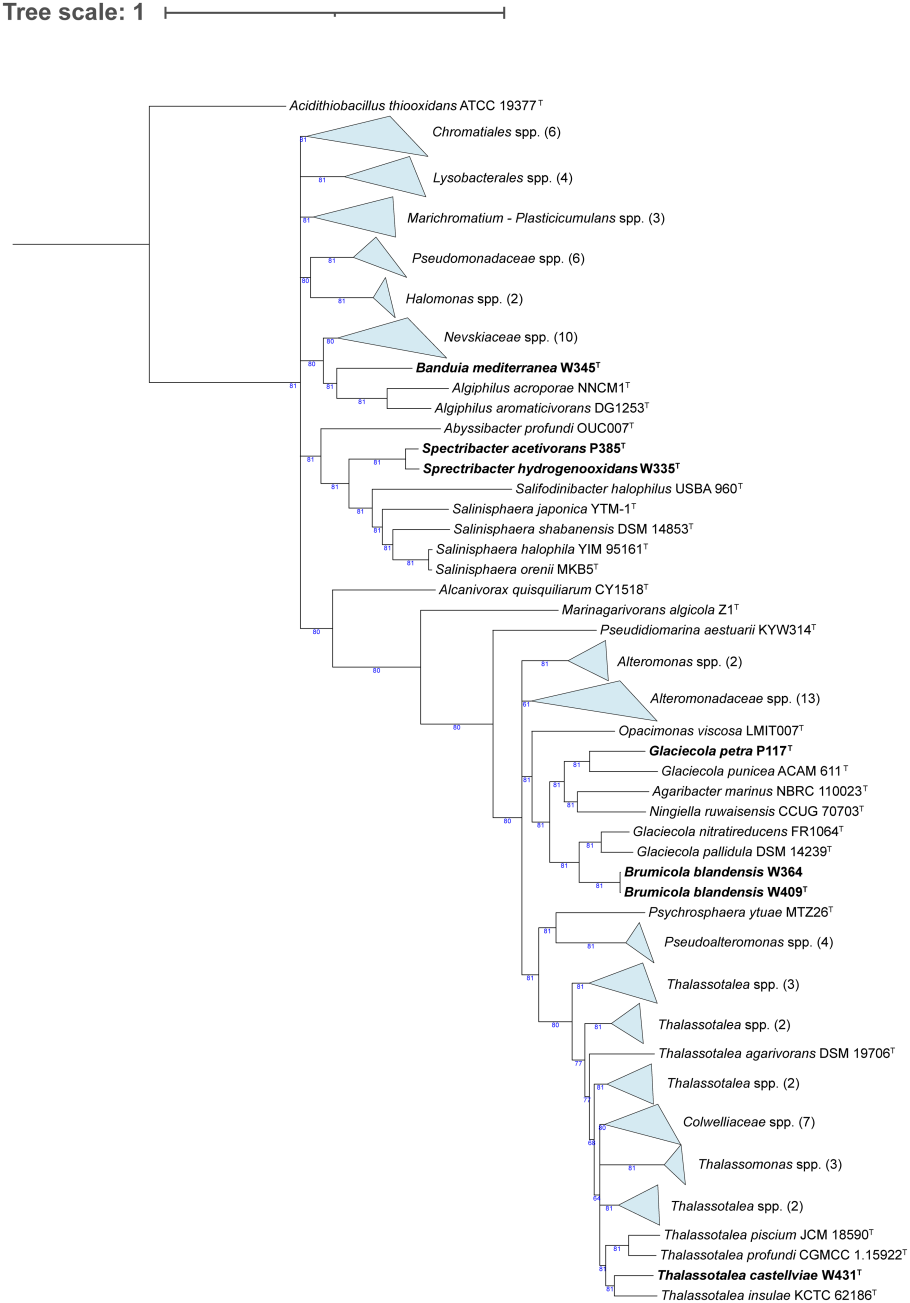
Phylogenomic tree generated with UBCG2 (Kim J, et al., 2021) of the strains belonging to the class *Gammaproteobacteria* and their closest neighbors according to the TYGS (Type Strain Genome Server). The strains characterized in this study are highlighted in bold. Accession numbers for these genomes can be found in Supplementary Table S5. The numbers at the nodes indicate the gene support index, with 81 being the maximum value.

We phylogenomically compared the genomes of the *Alphaproteobacteria*, where strain F158^T^ formed a tree branch close to *Tropicimonas isoalkanivorans* DSM 19548^T^ and *Tropicimonas sediminicola* DSM 29339^T^ inside a node within members of the *Roseobacteraceae* family (Supplementary Table S5; Figure 1). Strain W311^T^ appeared in a node with other *Stakelama* species, having *Stakelama marina* LXI357^T^ as its closest neighbor. Finally, strain F390^T^ formed a relatively long branch in a node shared with *Croceicoccus hydrothermalis* JLT 1^T^, which in turn was included in another node with five other *Croceicoccus* species. In the case of *Gammaproteobacteria* (Figure 2), strain W345^T^ formed a reasonably long branch inside a clade between members of the *Nevskiaceae* family and two *Algiphilus* species, which were together in a different node. Strains P385^T^ and W335^T^ constituted a different node inside a clade with members of the family *Salinispheraceae*. Strain P117^T^ formed a node with *Glaciecola punicea* ACAM 611^T^ in the same clade as strains W364 and W409^T^, which grouped together in a node related to different one which contains *Glaciecola nitratireducens* FR1064^T^ and *Glaciecola pallidula* DSM 14239^T^. In the case of strain W431^T^, it was placed among other *Thalassotalea* species, grouping with *Thalassotalea insulae* KCTC 62186^T^.

**Figure 3:**
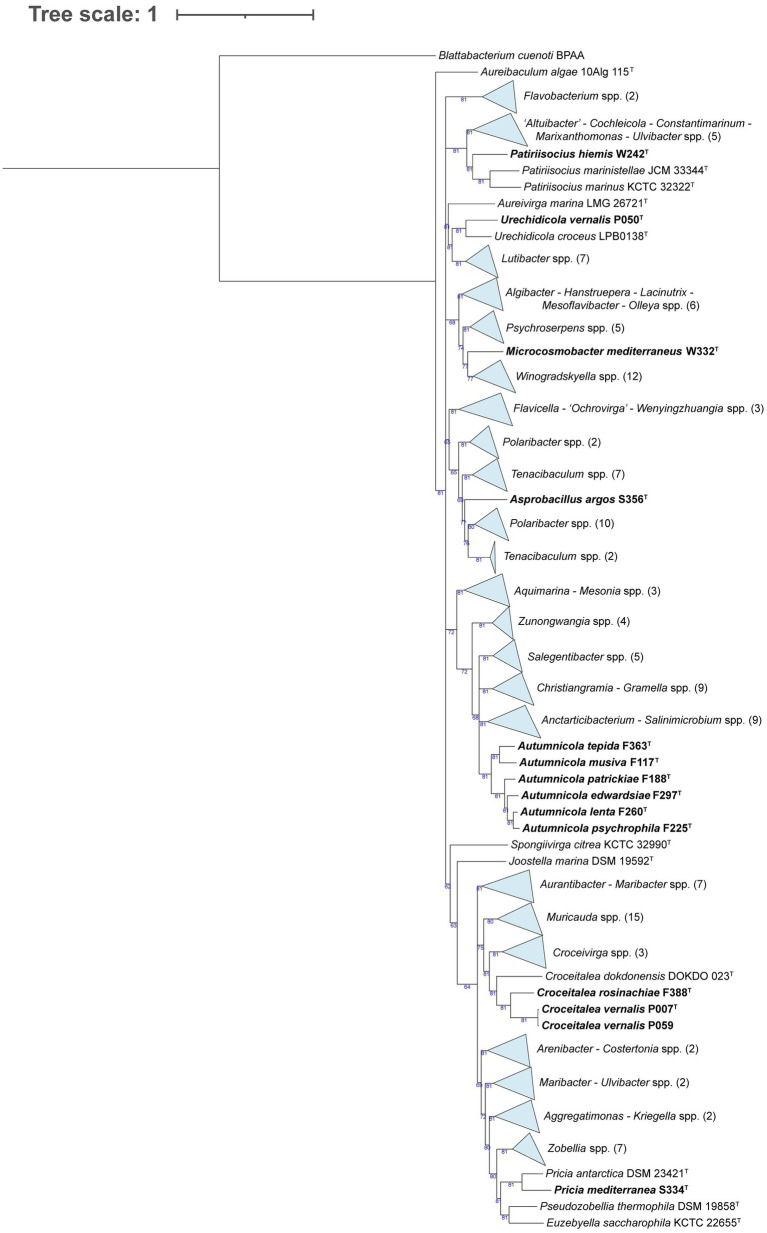
Phylogenomic tree generated with UBCG2 (Kim J, et al., 2021) of the strains belonging to the family *Flavobacteriaceae* and their closest neighbors according to the TYGS (Type Strain Genome Server). The strains characterized in this study are highlighted in bold. Accession numbers for these genomes can be found in Supplementary Table S5. The numbers at the nodes indicate the gene support index, with 81 being the maximum value.

**Figure 4:**
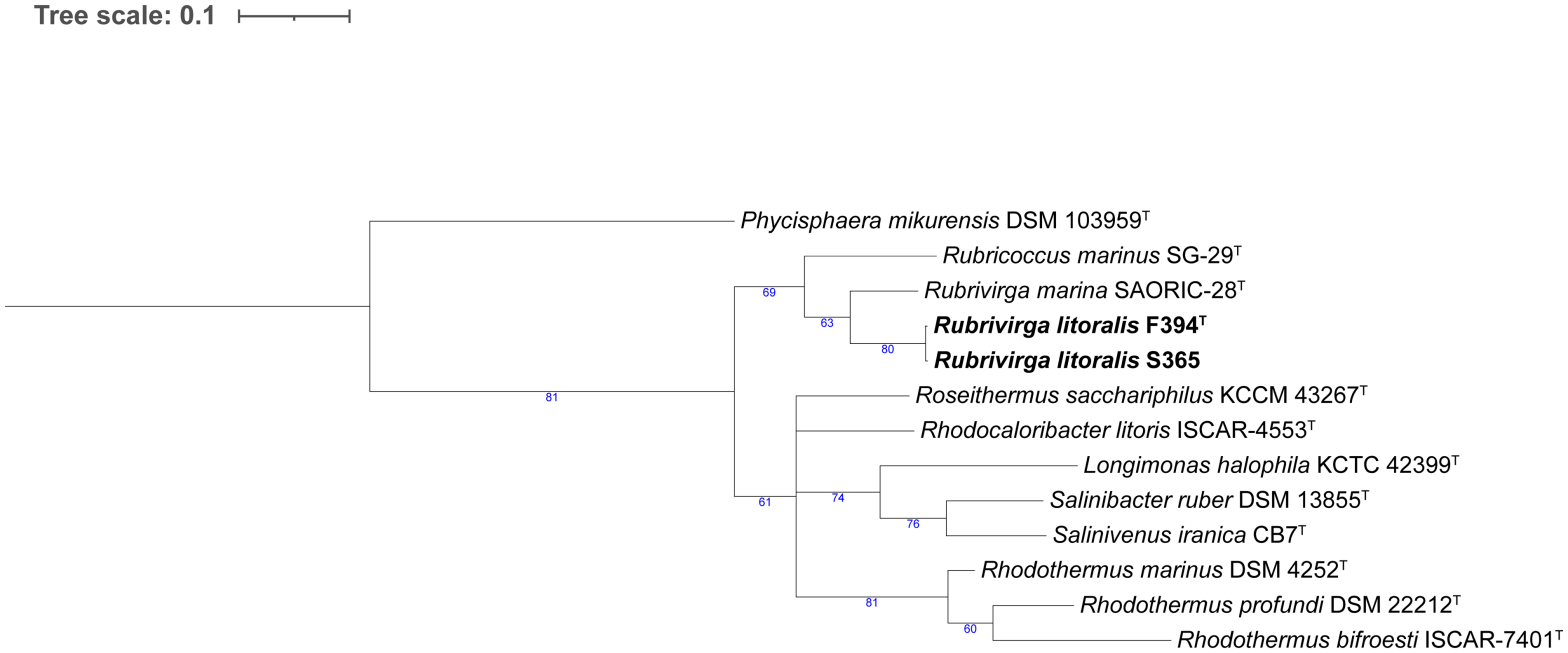
Phylogenomic tree generated with UBCG2 (Kim J, et al., 2021) of the strains belonging to the class *Rhodothermia* and their closest neighbors according to the TYGS (Type Strain Genome Server). The strains characterized in this study are highlighted in bold. Accession numbers for these genomes can be found in Supplementary Table S5. The numbers at the nodes indicate the gene support index, with 81 being the maximum value.

In the *Flavobacteriaceae* tree (Figure 3), strain W242^T^ comprised a branch close to a node containing two *Patiriisocius* species. Strain P050^T^ groups with *Urechidicola croceus* LBP0138^T^. Strain W332^T^ was placed in a node that contained 12 *Winogradskyella* species. In the case of strain S356^T^, it formed a single, long root inside a clade that included several *Polaribacter* and *Tenacibaculum* species. Strains F363^T^, F117^T^, F188^T^, F297^T^, F260^T^, and F225^T^ formed a clearly differentiated group in the tree. Strains F388^T^, P007^T^, and P059 also grouped together, close to a root formed by *Croceitalea dokdonensis* DOKDO 023^T^. Finally, strain S334^T^ was grouped with *Pricia antarctica* DSM 23421^T^. The last tree, formed by species of *Rhodothermia* (Figure 4), placed strains F394^T^ and S365 in a node with *Rubrivirga marina* SAORIC-28^T^.

### 3.3 Physiology and chemotaxonomy

All strains were Gram-negative, oxidase-positive, and catalase-positive, except for P050^T^, which was catalase-negative. Flexirubin pigmentation was not detected in any of the strains. The isolates varied in their time of growth to colony detection and maximal biomass density in MA; those pertaining to the *Alteromonadaceae* family were the most reactive, taking 1–2 days to create visible colonies. They were followed by *Flavobacteriaceae* strains W242^T^, F363^T^, F117^T^, F188^T^, F297^T^, F225^T^, and S334^T^; and *Alphaproteobaceria* strains F158^T^ and W311^T^, which presented a growth time to detection of 2–4 days. Strains F388^T^, P007^T^, and P059 (closely related to *Croceitalea* species) took 4 days to grow and had short viabilities. Strain P117^T^ also took around 4 days to grow, always in the form of very solid, stone-like colonies, and also presented a short viability. The slowest strains, taking 6–7 days to grow, were the *Flavobacteriaceae* P050^T^, W332^T^, S356^T^, and F260^T^; the *Alphaproteobacteria* F390^T^; the *Gammaproteobacteria* W345^T^, P385^T^, and W335^T^; and the *Rhodothermia* F394^T^ and S365. Strain W345^T^ formed small, flat, and transparent colonies after 6–7 days of growth, but the colonies became bigger, more mucous, and opaque-white over time, while isolates W335^T^ and P385^T^ presented small, transparent, and very thin colonies. Strains F394^T^ and S365 also resulted in small colonies. All the strains could grow in mR2A except F388^T^, P007^T^, P059, P050^T^, W332^T^ (*Flavobacteriaceae*), P117^T^ (*Gammaproteobacteria*), F394^T^, and S365 (*Rhodothermia*).

The main phenotypic characteristics of the strains used in this study are presented in Table 2. Notably, strains F188^T^, F225^T^, W364, W409^T^, and W431^T^ grew at 4°C. All strains except F388^T^, W332^T^, and P117^T^ grew at 15°C, and all of them grew at 28°C. At 37°C, the results were negative for some *Flavobacteriaceae* and *Alteromonadaceae* strains, and at 40°C, only strain F363^T^ presented growth. None of the strains grew in 0% (w/v) sea salts or in 2% (w/v) NaCl as the only salt. Some isolates had a broad salinity range: F158^T^, F188^T^, F297^T^, F260^T^, F225^T^ (closely related among them), S356^T^, and P385^T^. On the contrary, strains P059, P050^T^, P117^T^, and W332^T^ only grew in 3–4% (w/v) sea salts, 3–5% in the case of P007^T^, and 1.5–3% for S356^T^. Additionally, isolates W311^T^, F390^T^, F363^T^, F117^T^, S334^T^, W345^T^, and W335^T^ could grow down to 0.5% (w/v) sea salts, but only W335^T^ grew to more than 6%. Strains F394^T^ and S365 (which are closely related to *Rubrivirga*) differed in their salt preference: while strain F394^T^ thrived in 3–6% (w/v) sea salts, strain S365 preferred 1.5–3%.

**Table 2:** Main phenotypic characteristics of the novel strains.

Part A										
Features	1	2	3	4	5	6	7	8	9	10
Cell morphology	Rods	Rods	Coccobacilli	Rods	Rods	Rods	Rods	Rods	Rods	Rods
Colony color	Pink	Yellow	Yellow	White	White	White	Cream	Cream	Cream	Cream
Motility	−	−	−	−	−	−	−	+	−	+
Catalase	+	+	+	+	+	+	+	+	+	+
Temperature range (°C)	15–37	15–37	15–28	15–37	15–37	15–37	15–28	4–37	4–37	4–37
Salinity range (% sea salts)	0.5–12	0.5–4	0.5–5	0.5–5	1.5–11	0.5–10	3–4	3–4	2–9	1.5–8
Hydrolyses of:										
Casein	−	−	−	−	−	−	NG	+	+	+
Starch	w	+	w	−	−	−	NG	+	+	+
Alginate	−	w	−	−	−	−	+	+	−	−
Tween 80	−	+	NG	−	NG	NG	NG	NG	NG	NG
DNA	−	−	−	−	−	NG	NG	+	+	NG
API 20NE:										
NO_2_ formation	−	−	−	−	−	−	−	−	−	+
N_2_ formation	−	−	−	−	−	−	−	−	−	−
TRP (indole formation)	−	−	−	−	−	−	−	−	−	−
GLU (glucose fermentation)	−	−	−	−	−	−	−	−	−	−
ADH (arginine dihydrolase)	−	−	−	−	−	−	−	−	−	−
URE (urease)	−	−	−	−	−	−	−	−	−	−
ESC (aesculin hydrolysis)	−	+	−	−	−	−	+	+	+	+
GEL (gelatin hydrolysis)	−	−	−	−	−	−	−	+	−	+
PNPG (β-galactosidase)	−	−	−	−	−	−	+	+	+	−
GLU (glucose assimilation)	+	+	+	+	+	−	+	+	−	+
ARA (arabinose assimilation)	+	+	+	+	+	+	+	+	+	+
MNB (mannose assimilation)	+	+	+	+	+	+	+	+	+	+
MAN (mannitol assimilation)	+	+	+	+	+	+	+	+	+	+
NAG (n-acetyl-glucosamine assimilation)	+	+	+	+	+	+	+	+	+	+
MAL (maltose assimilation)	+	+	−	+	+	−	+	+	+	+
GNT (gluconate assimilation)	+	+	+	+	+	+	+	+	+	+
CAP (capric acid assimilation)	+	−	+	+	+	−	+	+	−	+
ADI (adipic acid assimilation)	+	+	+	+	+	+	+	+	+	+
MLT (malate assimilation)	+	+	+	+	+	+	+	+	+	+
CIT (citrate assimilation)	−	−	−	−	−	−	−	−	−	−
PAC (phenylacetic acid assimilation)	−	+	+	+	+	+	+	+	+	−

Part B																
Features	11	12	13	14	15	16	17	18	19	20	21	22	23	24	25	26
Cell morphology	Rods/chains	Rods/chains	Rods/chains	Rods	Rods/chains	Rods/chains	Rods/chains	Rods/chains	Rods/chains	Rods	Rods/chains	Rods/chains	Rods/chains	Rods	Peanuts	Peanuts
Colony color	Yellow	Yellow	Yellow–orange	White	Yellow	Yellow–orange	Yellow	Yellow	Yellow	Yellow	Orange	Orange	Orange	Orange	Red	Red
Motility	−	−	−	−	−	−	−	−	−	−	−	−	−	−	−	−
Catalase	+	−	+	+	+	+	+	+	+	+	+	+	+	+	+	+
Temperature range (°C)	15–37	4–28	28	15–28	15–40	15–37	4–37	15–28	15–28	4–37	28	15–28	15–28	15–28	15–37	15–37
Salinity range (% sea salts)	2–8	3–4	3–4	1.5–3	0.5–4	0.5–7	1.5–11	1–11	1.5–11	0.5–11	2–5	3–5	3–4	0.5–5	3–6	1.5–3
Hydrolyses of:																
Casein	+	−	+	NG	+	w	−	−	+	+	NG	NG	−	+	NG	NG
Starch	−	+	−	+	w	w	w	w	w	w	NG	−	−	−	+	+
Alginate	−	−	−	NT	−	−	−	−	−	−	+	+	+	NT	−	NT
Tween 80	NG	NG	NG	NG	+	NG	NG	NG	NG	NG	NG	NG	NG	+	NG	NG
DNA	+	NG	NG	NT	+	+	+	+	+	+	NG	NG	NG	NT	NG	NT
API 20NE:																
NO_2_ formation	−	−	−	NT	−	−	−	−	−	−	−	−	−	−	+	+
N_2_ formation	−	−	−	NT	−	−	−	−	−	−	−	−	−	−	−	−
TRP (indole formation)	−	−	−	NT	−	−	−	−	−	−	−	−	−	−	−	−
GLU (glucose fermentation)	−	−	−	NT	−	−	−	−	−	−	−	−	−	−	−	−
ADH (arginine dihydrolase)	−	−	−	NT	−	−	−	−	−	−	−	−	−	−	−	−
URE (urease)	−	−	−	NT	−	−	−	−	−	−	−	−	−	−	−	−
ESC (aesculin hydrolysis)	−	−	−	NT	+	+	+	+	+	+	+	+	+	+	+	+
GEL (gelatin hydrolysis)	+	−	−	NT	+	−	−	−	−	−	−	−	−	−	−	−
PNPG (β-galactosidase)	−	−	−	NT	+	−	+	−	−	+	+	−	−	+	−	+
GLU (glucose assimilation)	+	+	+	NT	+	+	+	+	+	+	−	+	+	−	+	−
ARA (arabinose assimilation)	+	+	+	NT	+	+	+	+	+	+	−	+	+	−	+	−
MNB (mannose assimilation)	+	+	+	NT	+	+	+	+	+	+	+	+	+	−	+	−
MAN (mannitol assimilation)	+	+	+	NT	+	+	+	+	+	+	+	+	+	−	+	−
NAG (n-acetyl-glucosamine assimilation)	+	+	+	NT	+	+	+	+	+	+	+	+	+	−	+	−
MAL (maltose assimilation)	+	+	+	NT	+	+	+	+	+	+	+	+	+	−	+	−
GNT (gluconate assimilation)	+	+	+	NT	.	+	+	−	+	+	+	+	+	−	+	−
CAP (capric acid assimilation)	+	+	+	NT	.	+	+	+	−	−	+	−	−	−	−	−
ADI (adipic acid assimilation)	+	+	+	NT	+	+	+	+	+	+	+	+	+	−	+	−
MLT (malate assimilation)	+	+	+	NT	+	+	+	+	+	+	+	+	+	−	−	−
CIT (citrate assimilation)	−	−	−	NT	−	+	−	−	+	−	−	−	−	−	−	−
PAC (phenylacetic acid assimilation)	−	+	+	NT	+	+	+	+	+	+	+	+	+	−	+	−

Interestingly, strains F388^T^, P007^T^, and P059 showed contrasting results in API ZYM strips: esterase activity was found only in P007^T^, and α-glucosidase activity was found in F388^T^ and P007^T^, but not in P059^T^. API 20NE assimilation assays were considered positive even when low turbidity was observed. Notably, strains F394^T^, S365 (*Rhodothermia*), and W431^T^ (related to *Thalassotalea* species) showed nitrite reductase activity. None of the strains showed glucose fermentation or urease activity. Aesculin hydrolysis was seen in most of the strains, and a few isolates hydrolyzed gelatin.

Most of the isolates presented in this study had weak or no growth in the positive control for carbon sources in a modified Basal Medium (Supplementary Table S6), making it challenging to reach conclusions based on this assay. F158^T^ was the strain that assimilated most carbon sources (28 out of 43, comprising sugars, organic acids, and amino acids). Aside from that, strains F363^T^, F117^T^, and W311^T^ showed a preference for sugars, while the *Gammaproteobacteria* isolates seemed to prefer organic acids and amino acids, although sometimes with weak growth or unclear results. L-leucine was the only compound where no growth by any of the strains could be detected.

The only polar lipid predicted from the genomes of all *Flavobacteriaceae* was phosphatidylethanolamine (PE), except strain S334^T^, which also contained diphosphatidylglycerol (DPG; Supplementary Table S7). All *Gammaproteobacteria* strains presented phosphatidylglycerol (PG) and PE, and W345^T^, P385^T^, and W335^T^ genomes also contained DPG. All *Alphaproteobacteria* strains had enzymes for DPG and PG synthesis. Apart from these, strain F158^T^ contained phosphatidylcholine (PC), strain F390^T^ had PE and phosphatidyl m-inositol (PI), and strain W311^T^ contained PI. Both *Rhodothermia* genomes present PE and PG synthesis enzymes. As for their respiratory quinones, all *Flavobacteriaceae* strains presented menaquinone 6 synthesis enzyme in their genomes; *Gammaproteobacteria* isolates had ubiquinone 8; *Alphaproteobacteria* possessed ubiquinone 10; and *Rhodothermia* genomes had menaquinone 7. A screening of gliding motility genes (*gld*) also indicated that most *Flavobacteriaceae* genomes contained all the *gld* genes, yet strains F388^T^, P007^T^, and P059 lacked *gldI*, while P050^T^, W332^T^, and S356^T^ lacked *gldI* and *gldH* (Supplementary Table S7). In addition, all *Gammaproteobacteria* strains except W364 and W409^T^ contained *gldF* and *gldA*. No *gld* genes were found in the *Alphaproteobacteria* or *Rhodothermia* genomes.

### 3.4 Biogeochemical relevance

**Figure 5:**
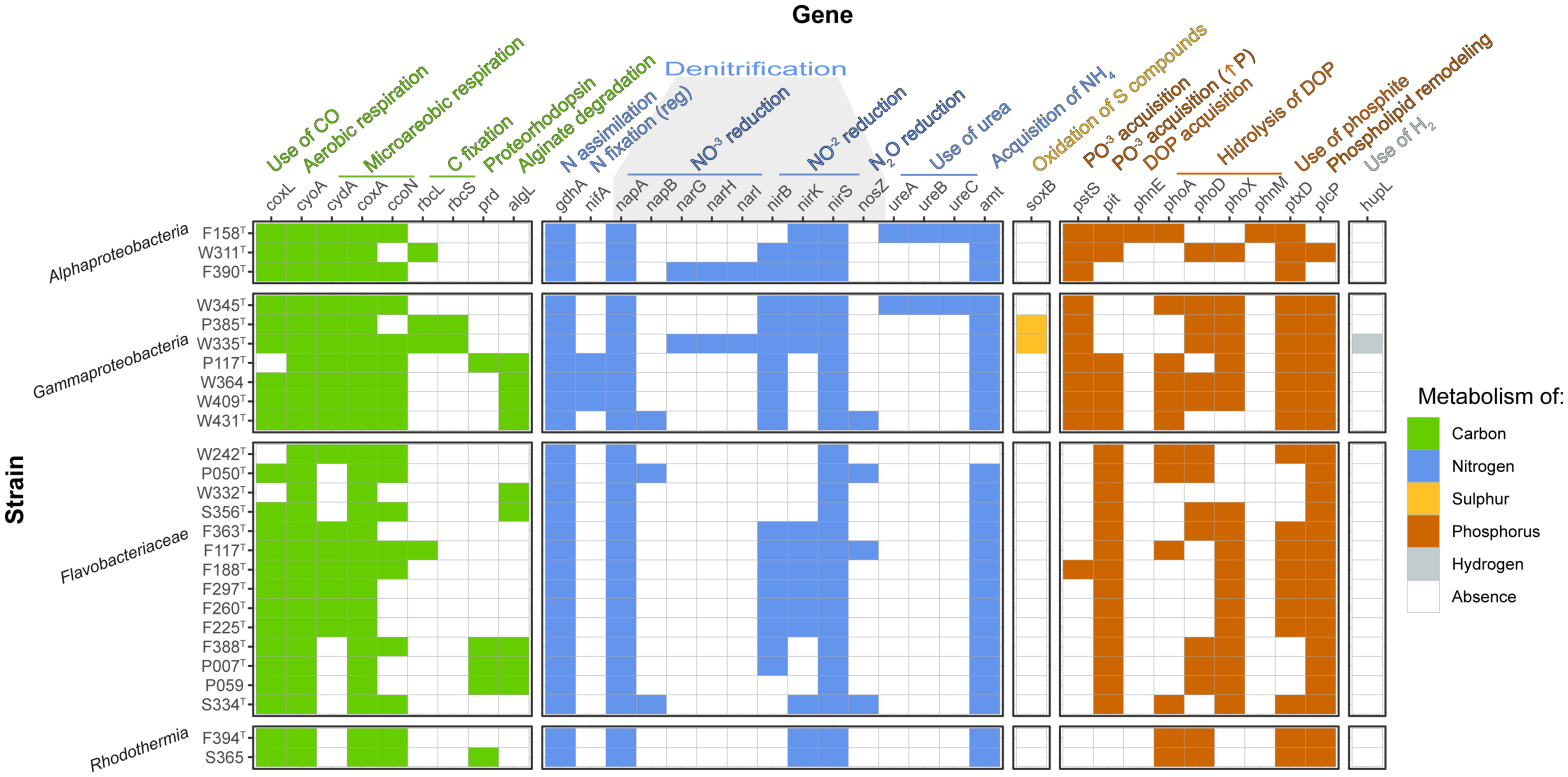
Heatmap indicating the presence/absence of genes relevant to biogeochemical cycles among the novel genomes. Only the genes with at least one positive value are shown.

Several genes involved in biogeochemical processes in the cycles of carbon, nitrogen, sulfur, phosphorus, iron, and hydrogen were present among the strains (Figure 5). Concerning the carbon cycle, most characterized strains possessed carbon-monoxide dehydrogenase (*coxL*), which would permit them to use CO as an energy source. The only exceptions are strains P117^T^, W242^T^, and W332^T^. Interestingly, the closely related strains P385^T^ and W335^T^ carried the genes *rbcL* and *rbcS*, which codify for the two subunits of the RuBisCO enzyme, a marker of carbon fixation. Strains P117^T^ (affiliated to *Glaciecola*), F388^T^, P007^T^, P059 (affiliated to *Croceitalea*), and S365 (affiliated to *Rubrivirga*) presented proteorhodopsin (*prd*), indicating that these strains can use light as a complementary energy source. Some *Gammaproteobacteria* (those affiliating with *Alteromonadaceae* family) and *Flavobacteriaceae* (strains W332^T^, S356^T^, F388^T^, P007^T^, and P059) presented alginate-degrading genes.

The *gdhA* gene encoding glutamate 4ehydrogenase was found in all the strains, while the *nifA* gene for regulation of nitrogen fixation was detected in the three strains related to *Glaciecola* (P117^T^, W364, and W409^T^), but none of them could be classified as nitrogen fixers since no other *nif* gene was found. In addition, no genes implied by nitrification were found. We did find several genes related to denitrification in the genomes, but none of them contained the complete pathway. There were, however, three strains that presented the two subunits of the periplasmic nitrate reductase (W431^T^, P050^T^, and S334^T^), two strains that contained the three subunits of the membrane nitrate reductase (F390^T^ and W335^T^), all strains included at least one nitrite reductase, and four strains (W431^T^, F117^T^, P050^T^, and S334^T^) contained the *nosZ* gene codifying for nitrous-oxide reductase. Three subunits of urease were found in strains F158^T^ and W345^T^, implying that they could use urea as a nitrogen source. Strain W242^T^ seemed to lack the *amt* ammonium transporter.

Additionally, strains P385^T^ and W335^T^ (closely related *Gammaproteobacteria*) presented the *soxB* gene in their genomes and therefore could use sulfur compounds as an energy source. While all *Alphaproteobacteria* and *Gammaproteobacteria* strains presented the inorganic phosphate transport *pstS* gene, *Flavobacteriaceae* strains had instead the *pit* gene for acquisition of inorganic phosphate when phosphorus is in high concentrations (F188^T^ had both genes), and the *Rhodothermia* strains had none. Strain F158^T^ was the only one which presented the phosphonate transport system gene *phnE*. Most genomes contained at least one alkaline phosphatase gene (*phoA*, *phoD*, and *phoX*), while the ones that lacked them all were strains F390^T^ and W332^T^. In addition, strains F188^T^, F297^T^, F260^T^, and F225^T^ only presented alkaline phosphatase *phoX* in their genomes. F158^T^ was the only strain where *phnM* was detected. All strains had phosphite dehydrogenase *ptxD*, except P050^T^, W332^T^, S356^T^, F388^T^, P007^T^, and P059. Additionally, all strains but F158^T^ and F390^T^ had the *plcP* gene for phospholipid remodeling under low phosphorus availability. Interestingly, strain W335^T^ presented the NiFe hydrogenase *hupL*, which would confer the ability to oxidize hydrogen to obtain energy.

### 3.5 Biogeography

**Figure 6:**
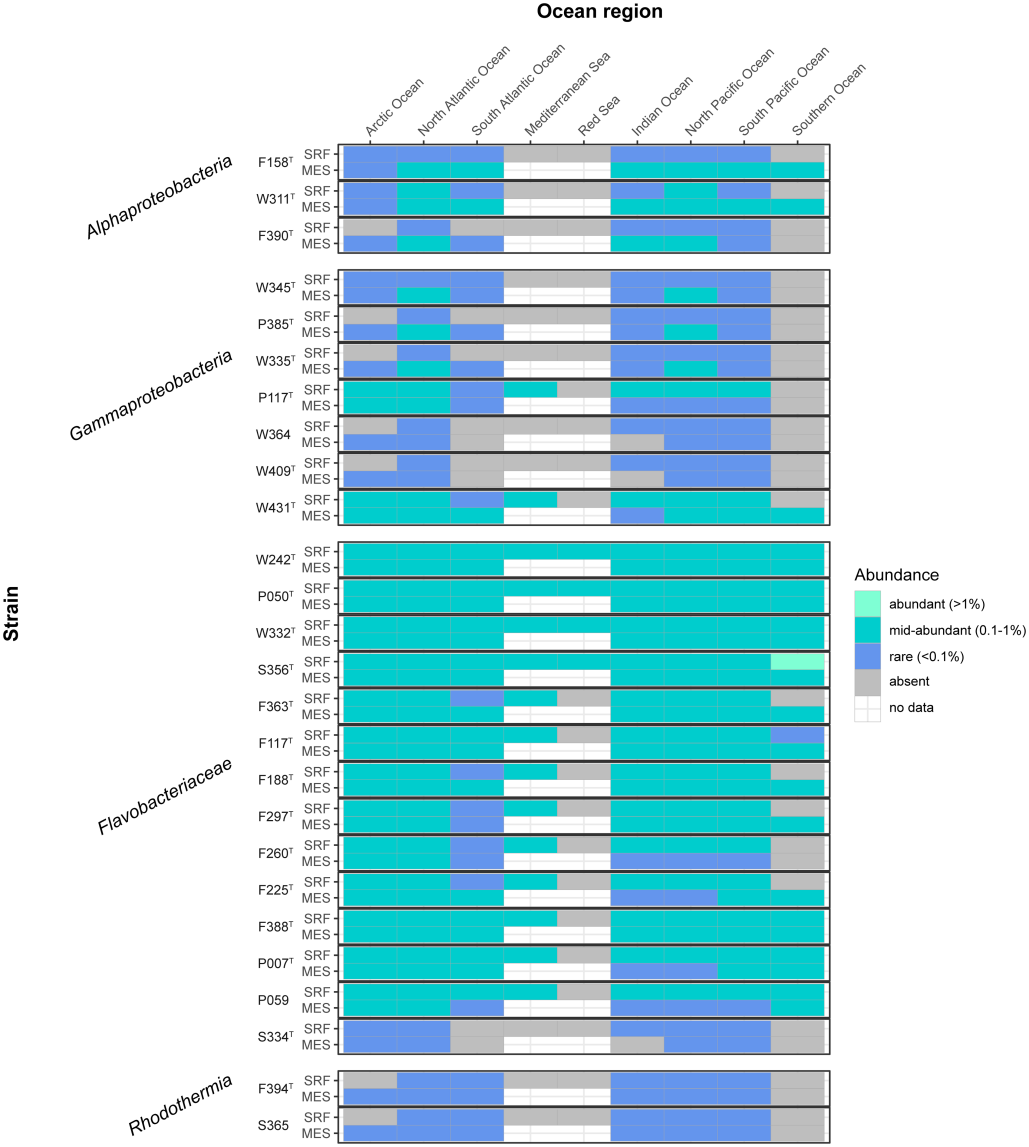
Heatmap indicating the abundance of the described strains in different oceans and layers. The gray color indicates absence, and the white color indicates no data. Data obtained by mapping the genomes to the Tara Oceans metagenomes. SRF, surface; MES, mesopelagic.

We mapped our genomes to the metagenomic reads sampled by the Tara Oceans expedition to generate a general picture of their presence in different areas and depths of the global oceans. Most of the novel strains here presented had medium relative contribution (0.1–1% of the reads) to the community in various ocean regions (Figure 6), especially the *Flavobacteriaceae* strains (except for S334^T^, which seemed to be rarer) and *Gammaproteobacteria* strains P117^T^ and W431^T^. Notably, strain S356^T^ reached a relatively high abundance (1.09%) in the Southern Ocean’s surface waters. The rarest strains, which did not surpass the rare biosphere threshold (<0.1% reads) in any region, were W364, W409^T^ (closely related strains from *Alteromonadaceae* family), F394^T^, and S365 (pertaining to the same species of *Rubrivirga*).

**Figure 7:**
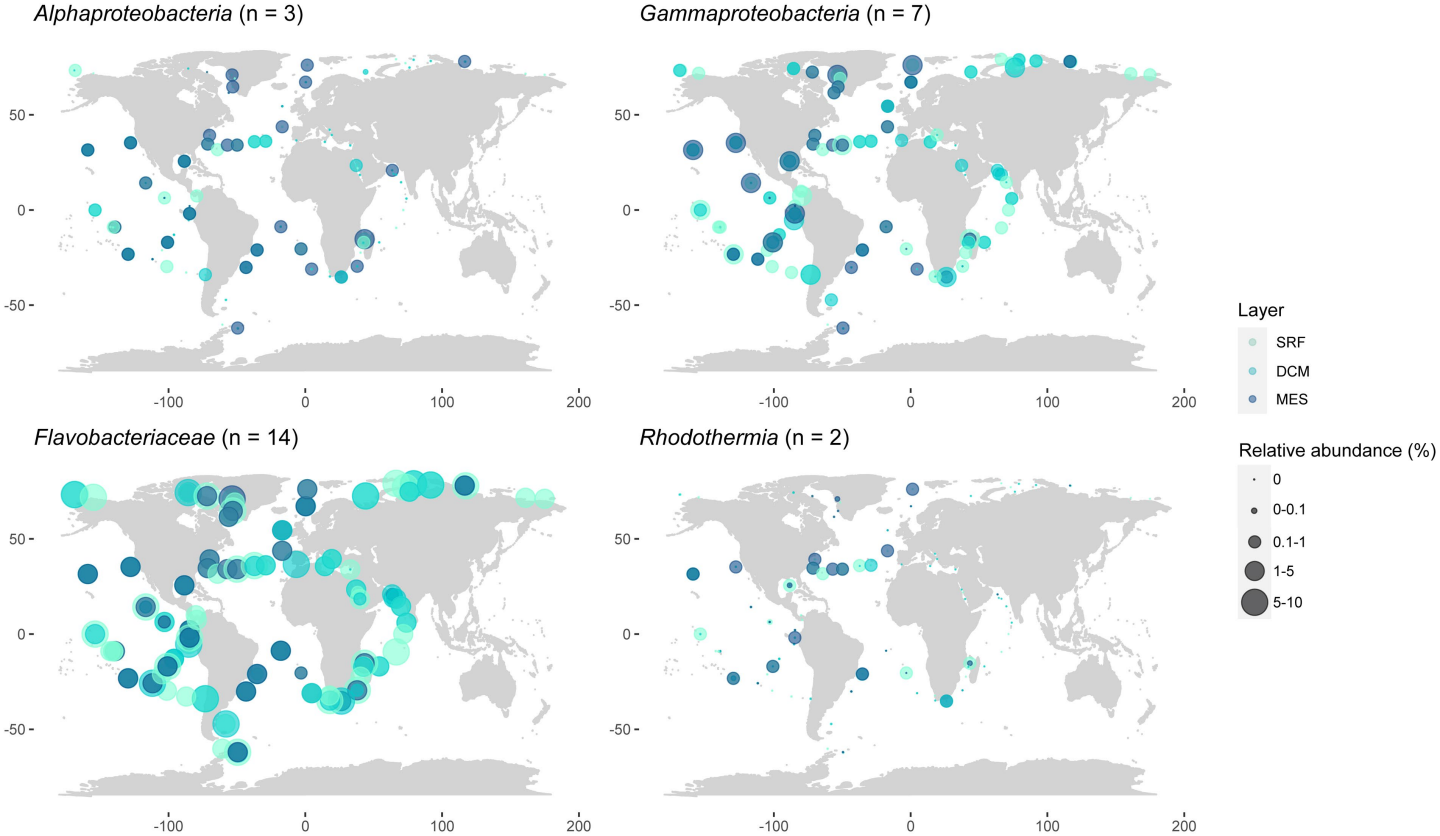
Geographic distribution of the presented strains grouped by class, taking into account different ocean layers. Data matched to the Tara Oceans metagenomes. SRF, surface; DCM, deep chlorophyll maximum; MES, mesopelagic.

As a whole, *Flavobacteriaceae* strains represented the most widespread and abundant group (they recruited up to 9.49% of the reads in the South Pacific Ocean’s DCM and represented 3.41 ± 1.89% of the total Tara Oceans reads), followed by *Gammaproteobacteria*, which were frequently more abundant in the mesopelagic (Figure 7), and had a maximum contribution of 2.08% of the reads in the South Pacific Ocean’s DCM and represented 0.46 ± 0.42% of the total dataset. *Alphaproteobacteria* strains, which summed 0.19 ± 0.29% of the total reads, were also present in most oceans with significant abundances, although the number of samples where they were detected was lower than the previous two groups. They contributed more to the mesopelagic, recruiting up to 1.32% of the reads in the Indian Ocean. The *Rhodothermia* strains, which represented only 0.03 ± 0.08% of the Tara Oceans reads, were the less abundant, and they were detected in fewer samples, but they were still present in all ocean regions except the Mediterranean and Red Seas and the Southern Ocean.

## 4 Discussion

We compared the phenotypic and genomic characteristics of the novel strains described above to those of their closest relatives in order to resolve their taxonomic classification. None of the new strains had >95% ANI nor >70% dDDH compared to their closest relatives, which confirms their novelty according to the commonly established thresholds (Meier-Kolthoff et al., 2013; Jain et al., 2018). However, there are three pairs of strains that score > 95% ANI between them. Thus, the 26 strains characterized here represent 23 novel marine bacterial species from four different classes: *Alphaproteobacteria*, *Gammaproteobacteria*, *Bacteroidia* (family *Flavobacteriaceae*), and *Rhodothermia*.

### 4.1 Differential characteristics of the *Alphaproteobacteria* strains

Strain F158^T^ has *Tropicimonas isoalkanivorans* DSM 19548^T^ and *Tropicimonas sediminicola* DSM 29339^T^ as its closest neighbors (Figure 1). *T. isoalkanivorans* is the type species of genus *Tropicimonas* (Harwati et al., 2009). Their DNA G + C content is similar: strain F158^T^ has 66.9 mol%, *T. isoalkanivorans* presents 66.5 mol%, and *T. sediminicola* has 68.5 mol%. They have the same major polar lipids (Supplementary Table S8), but while strain F158^T^ and *T. sediminicola* (Shin et al., 2012) are catalase positive and non-motile, *T. isoalkanivorans* is catalase negative and motile. The temperature and salinity ranges also differ: strain F158^T^ grows between 15°C and 37°C and has a broad salinity range (0.5–12% (w/v) sea salts) but cannot grow without salts, *T. sediminicola* grows at 15–42°C and can thrive in 0–8% (w/v) NaCl, and *T. isoalkanivorans* grows between 10–46°C and 1–6% (w/v) NaCl. Taking into account the low AAI values that strain F158^T^ has with its closest neighbors (67.06% with *T. isoalkanivorans* DSM 19548^T^ and 66.82% with *T. sediminicola* DSM 29339^T^) and that they are distinct in several phenotypic traits (Supplementary Table S8), it could be considered that this strain represents a novel genus (Nicholson et al., 2020). However, given their placement in the phylogenomic tree, the fact that this genus is already phenotypically diverse, and that they do share some core characteristics (major respiratory quinones, major polar lipids, a highly similar G + C content, and several common substrates), we propose for strain F158^T^ a novel species with the name *Tropicimonas omnivorans*.

Strain W311^T^ has *Stakelama marina* LXI357^T^ as its closest neighbor and is also close to *Stakelama pacifica* DSM 25059^T^ (Figure 1), the type species of genus *Stakelama* (Chen et al., 2010). The three strains have very similar G + C content: W311^T^ has 65.8 mol%, *S. pacifica* 66 mol%, and *S. marina* 64.1 mol% (Wang et al., 2023), but they present some differences in their major polar lipids (Supplementary Table S8). While W311^T^ and *S. marina* are non-motile, *S. pacifica* presents motility. Their temperature and salinity ranges are similar: strain W311^T^ survives from 15°C to 37°C and in 0.5–4% (w/v) sea salts, *S. pacifica* does so in 5–37°C and 0–5% (w/v) NaCl, and *S. marina* is the most different one, growing between 20–45°C and 0.5–11% (w/v) NaCl. Taking into account these traits, their common and differential assimilated carbon sources and enzymatic activities (Supplementary Table S8), the topology of the phylogenomic tree, and the relatively high AAI values between these strains, especially for *S. marina* LXI357^T^ (78.8% AAI), we propose for strain W311^T^ a novel species with the name *Stakelama saccharophila*.

Strain F390^T^ groups close to *Croceicoccus hydrothermalis* JLT1^T^ in the same clade as the type species, *Croceicoccus marinus* (Xu et al., 2009; Figure 1). The G + C content is more similar between strain F390^T^ (60.7 mol%) and *C. hydrothermalis* (63.2 mol%; Chen et al., 2022) than with *C. marinus* (71.5 mol%), and the same trend applies for their polar lipids (Supplementary Table S8). Contrary to strain F390^T^ and *C. hydrothermalis*, *C. marinus* is motile. Temperature ranges for *C. hydrothermalis* (5–45°C) and *C. marinus* (4–42°C) are broader than for strain F390^T^ (15–28°C). Similarly, strain F390^T^ thrives in 0.5–5% (w/v) sea salts, but *C. hydrothermalis* does from 0 to 12% (w/v) NaCl, and *C. marinus* grows in 0–10% (w/v) NaCl. Only strain F390^T^ is oxidase positive. In terms of carbon source utilization and enzymatic activities, strain F390^T^ is more similar to *C. marinus* than to *C. hydrothermalis* (Supplementary Table S8). Taking into account all these common and differential characteristics, the topology of the phylogenomic tree (Figure 1), and the AAI values (74.11% against *C. hydrothermalis* JLT1^T^ and 70.21% against *C. marinus* E4A9^T^), we propose for strain F390^T^ a novel species with the name *Croceicoccus esteveae*.

### 4.2 Differential characteristics of the *Gammaproteobacteria* strains

Strain W345^T^ shares a node with *Algiphilus acroporae* NNCM1^T^ and *Algiphilus aromaticivorans* DG1253^T^ (Figure 2). The G + C content of strain W345^T^ (64 mol%), *A. acroporae* (63.3 mol%; Sun et al., 2022), and *A. aromaticivorans* (63.6 mol%; Gutierrez et al., 2012) is quite similar. The main polar lipids of the three strains are mostly the same (Supplementary Table S8). We did not observe motility in strain W345^T^, but the two *Algiphilus* species are described as motile. Temperature ranges are similar: strain W345^T^ grows at 15–37°C, *A. acroporae* grows between 18°C and 37°C, and *A. aromaticivorans* has a broader range: from 4°C to 37°C. While strain W345^T^ survives between 0.5 and 5% (w/v) sea salts, with a weak growth in 6%, *A. acroporae* grows in 0.5–12% (w/v) NaCl, and *A. aromaticivorans* does so from 0 to 12% (w/v) NaCl, and thus, it does not require salts for growth. These strains have certain similarities but are quite different in terms of their substrate utilization and extracellular hydrolyses (Supplementary Table S8). In view of these facts and the low AAI between strain W345^T^ and *A. acroporae* NNCM1^T^ (61.31%) and *A. aromaticivorans* DG1253^T^ (60.33%), we propose for strain W345^T^ a novel genus and species with the name *Banduia mediterranea*.

Strains P385^T^ and W335^T^ group together in the phylogenomic tree (Figure 3) and have *Salinisphaera orenii* MKB5^T^ and *Salinisphaera halophila* YIM 95161^T^ as their closest neighbors in terms of AAI. Strains P385^T^ (65.7 mol%) and W335^T^ (65.6 mol%) have almost identical G + C content, while *S. halophila* (69.5 mol%; Zhang et al., 2012) and *S. orenii* (63.5 mol%; Park et al., 2012) flank them on the upper and lower side, respectively. Strains P385^T^ and W335^T^ have the same major polar lipids and are oxidase positive, but, in contrast, *S. halophila* contains PC and is oxidase negative (Supplementary Table S8). While we did not observe motility in strains P385^T^ or W335^T^, both *Salinisphaera* spp. are motile. The temperature ranges of the four strains are quite similar; strains P385^T^ and W335^T^ grow at 15–37°C, *S. halophila* at 15–40°C, and *S. orenii* has the broadest range at 10–40°C. In terms of salinity ranges, strains P385^T^ (1.5–11% w/v sea salts with weak results in 1%) and W335^T^ (0.5–8% w/v salts with weak growth in 9 and 10%) have a much lower tolerance than *S. halophila* (5–29% w/v NaCl) and *S. orenii* (5–25% w/v NaCl). Strains P385^T^ and W335^T^ share most enzymatic activities and carbon sources, but they present several differences with *S. orenii* (especially) and *S. halophila* (Supplementary Table S8). Considering these characteristics, the low AAI values (around 64%) that both strains share with their closest neighbors (Supplementary Table S5), and the topology of the phylogenomic tree, we propose for strains P385^T^ and W335^T^ a new genus and two novel species with the names *Spectribacter acetivorans* and *Spectribacter hydrogenooxidans*, respectively.

Strain P117^T^ clusters with the type species of genus *Glaciecola*, *G. punicea* ACAM 611^T^ (Bowman et al., 1998), and has *Ningiella ruwaisensis* CCUG 70703^T^ as its second closest strain (Figure 2). While strain P117^T^ has 39.9 mol% G + C, *G. punicea* contains 45.1 mol% (Bowman et al., 1998), and *N. ruwaisensis* has 43.2 mol% (Fotedar et al., 2020). Contrary to strain P117^T^, *G. punicea* and *N. ruwaisensis* are motile. The temperature ranges of strain P117^T^ and *G. punicea* are similar: 15–28°C and 15–25°C, respectively, but *N. ruwaisensis* has a broader range (15–45°C). The salinity range of strain P117^T^ is quite narrow (3–4% (w/v) sea salts), while *N. ruwaisensis* develops in 0–6% (w/v) NaCl. Strain P117^T^ has more common carbon sources and enzymatic activities with *G. punicea* than with *N. ruwaisensis* (Supplementary Table S8). Strain P117^T^ presents an AAI of 70.92% with *G. punicea* ACAM 611^T^ and 67.84% with *N. ruwaisensis* CCUG 70703^T^. With these values, strain P117^T^ could be proposed as a novel genus, but considering that in the phylogenomic tree (Figure 2) this strain clearly clusters with *G. punicea*, the type species of the genus *Glaciecola*, and in the absence of the type strain genome for some species of the genus to better assess its taxonomic status, we propose for strain P117^T^ a new species of the genus *Glaciecola* with the name *Glaciecola petra*.

In the same section of the tree (Figure 2), we find strains W364 and W409^T^, which share a node with *Glaciecola nitratireducens* FR1064^T^ and *G. pallidula* DSM 14239^T^. Strains W364 and W409^T^ have 99.25% ANI between them, and thus, they belong to the same species (Jain et al., 2018). Both strains present 42.6 mol% G + C, while *G. nitratireducens* (Baik et al., 2006) has 44 mol% and *G. pallidula* (Bowman et al., 1998) contains 40 mol%. While strain W364, *G. nitratireducens*, and *G. pallidula* are motile, we did not observe this in strain W409^T^. The temperature range of the novel strains is 4–37°C, although strain W364 presented weak growth at that maximum temperature. In contrast, *G. nitratireducens* grows between 15°C and 30°C, and *G. pallidula* develops at 10–20°C. Strains W364, W409^T^, and *G. nitratireducens* do so in 2–9% (w/v) sea salts. Most carbon sources and enzymatic activities displayed by strains W364 and W409^T^ are the same, and they are partly shared with *G. nitratireducens* and *G. pallidula*, with certain differences (Supplementary Table S8). Looking at the phylogenomic tree (Figure 2), it is noteworthy that *G. nitratireducens* and *G. pallidula* cluster with strains W364 and W409^T^ rather than with *G. punicea*, the type species of the genus *Glaciecola*. Moreover, *Agaribacter marinus* and *Ningiella ruwaisensis* are closer to *G. punicea*, making a polyphyletic branch. We wanted to explore this in more detail, using the Kostas Lab ANI/AAI matrix tool (Rodriguez-R and Konstantinidis, 2016) to compute an AAI matrix with all these strains and P117^T^. The matrix showed AAI values of 64–65% when comparing *G. punicea* ACAM 611^T^ to *G. nitratireducens* FR1064^T^ and *G. pallidula* DSM 14239^T^, while these two had 73–74% AAI with strains W364 and W409^T^ (Supplementary Figure S1). Strains W364 and W409^T^ also have a low AAI (63%) with *G. punicea* ACAM 611^T^. In view of all these data, we propose the reclassification of *Glaciecola pallidula* and *Glaciecola nitratireducens* into a new genus, resulting in the new combinations *Brumicola pallidula* and *Brumicola nitratireducens*, and for strain W409^T^, a new species with the name *Brumicola blandensis*.

At the bottom of the phylogenomic tree (Figure 2), we observe strain W431^T^ clustering with *Thalassotalea insulae* KCTC 62186^T^ and, more distantly, with the type species of the genus, *T. ganghwensis* (Zhang et al., 2014). Strain W431^T^ has a lower G + C (37.2 mol%) than *T. insulae* (41.3 mol%; Park et al., 2018) and *T. ganghwensis* (42 mol%). The temperature ranges of strain W431^T^ (4–37°C), *T. insulae* (10–37°C), and *T. ganghwensis* (15–40°C) are quite similar. The salinity ranges of strain W431^T^ and *T. ganghwensis* are almost identical (1.5–8% (w/v) sea salts and 1–8% (w/v) NaCl, respectively), while *T. insulae* can grow without salt and up to 6% (w/v) NaCl. Strain W431^T^ displays certain differences with *T. insulae* and *T. ganghwensis* in terms of assimilation of carbon sources and enzymatic activities (Supplementary Table S8). With this information, the phylogenomic tree that clearly places W431^T^ within the *Thalassotalea* species (Figure 2) and the AAI values (Supplementary Table S5), we propose for strain W431^T^ a new species with the name *Thalassotalea castellviae*.

### 4.3 Differential characteristics of the *Flavobacteriaceae* strains

As it is usual in the *Flavobacteriaceae* (Bernadet et al., 2002), all novel strains from that family had PE as their major polar lipid, menaquinone 6 as their predominant respiratory quinone, and are non-motile. In addition, they did not present flexirubin-type pigments, which are common in marine strains from this family (Bernadet et al., 2002). Thus, unless there is a difference between the novel strains and those used for the comparisons, these characteristics will not be mentioned below.

Strain W242^T^ is placed close to *Patiriisocius marinistellae* JCM 33344^T^ and *P. marinus* KCTC 32322^T^ in the phylogenomic tree (Figure 3). They are the only two characterized species of that recent genus, *P. marinistellae*, its type species (Kawano et al., 2020). The three strains have very similar G + C content: 33.7 mol% for W242^T^, 33.4 mol% for *P. marinistellae* (Kawano et al., 2020), and 33.3 mol% for *P. marinus*. Strain W242^T^ (15–37°C), *P. marinistellae* (4–30°C), and *P. marinus* (4–35°C) have relatively similar temperature ranges, and while strain W242^T^ develops in 2–8% (w/v) sea salts, the others do it in 0.5–5% (w/v) NaCl. Although strain W242^T^ differs in several carbon sources and enzymatic activities from *P. marinus* and *P. marinistellae* (Supplementary Table S8), in view of their clear placement in the phylogenomic tree (Figure 3), and also considering the AAI values (Supplementary Table S5), we propose for strain W242^T^ a new species with the name *Patiriisocius hiemis*.

Strain P050^T^ clearly clusters with *Urechidicola croceus* (Figure 3), the only species of that genus to date (Shin and Yi, 2020). Strain P050^T^ has a higher G + C content (33.5 mol%) than *U. croceus* (30.4 mol%). Their temperature ranges are almost identical: 4–28°C for strain P050^T^ and 4–28°C for *U. croceus*, and while strain P050^T^ has a narrow salinity range (only 3–4% (w/v) sea salts), *U. croceus* can grow between 1 and 5% (w/v) NaCl. Strain P050^T^ is oxidase-positive and catalase-negative, while *U. croceus* presents the opposite profile. Both strains coincide in their capacity to hydrolyze DNA and their negative results in indole production and citrate assimilation in API 20NE. However, strain P050^T^ was also positive (in API 20NE strips but not on agar plates) in the assimilation of several compounds for which *U. croceus* gave negative results (Supplementary Table S8). Another difference is that *U. croceus* reduces nitrate to nitrite, but strain P050^T^ does not. In view of these data, the AAI values in Supplementary Table S5, and the topology of the phylogenomic tree, we propose for strain P050^T^ a new species with the name *Urechidicola vernalis*.

Following the *Flavobacteriaceae* phylogenomic tree, strain W332^T^ shares a node with 12 *Winogradskyella* species, from which it is separated by a relatively long branch (Figure 3). It is close to *W. echinorum* but also quite close to the type species *W. thalassocola* (Nedashkovskaya et al., 2005). Strain W332^T^ has 32.5 mol% G + C, *W. thalassocola* presents 34.6 mol%, and *W. echinorum* has 33.6 mol% (Nedashkovskaya et al., 2009). While the temperature (28°C) and salinity ranges (3–4% (w/v) sea salts) of strain W332^T^ are pretty narrow, *W. thalassocola* develops at 4–33°C and 1–8% (w/v) NaCl, and *W. echinorum* grows at 4–37°C and in 1–6% (w/v) NaCl. For those characteristics for which data exists, some differences between strain W332^T^, *W. echinorum*, and *W. thalassocola* in terms of assimilated carbon sources and enzymatic activities could be identified (Supplementary Table S8). Considering that strain W332^T^ is relatively distant to its closest *Winogradskyella* neighbor (72.62% AAI to *W. echinorum*; Supplementary Table S5) and that it is differentiated from all the *Winogradskyella* strains by a long branch in the phylogenomic tree (Figure 3), we propose for strain W332^T^ a new genus and species with the name *Microcosmobacter mediterraneus*.

Strain S356^T^ lies between several *Tenacibaculum* and *Polaribacter* spp., with *Polaribacter septentrionalilitoris* ANORD1^T^ and *P. pectinis* L12M9^T^ as its closest neighbors. Strain S356^T^ has a G + C content (35.2 mol%) more similar to *P. pectinis* (36.4 mol%) than to *P. septentrionalilitoris* (30.6 mol%), and it has a narrow temperature range (15–28°C) compared to its relatives (4–25°C for *P. pectinis*, 0–45°C for *P. septentrionalilitoris*). While strain S356^T^ grows only on 1.5–3% (w/v) sea salts, *P. pectinis* grows in 0.5–6% (w/v) NaCl and *P. septentrionalilitoris* in 2–10% (w/v) NaCl. *P. pectinis* and *P. septentrionalilitoris* assimilate more compounds and have more positive results for enzymatic activities than strain S356^T^ (Supplementary Table S8). Given the evident phenotypic differences and the long branch formed by strain S356^T^ on the phylogenomic tree (Figure 3), we explored the AAI values of this genome with its closest neighbors using the ANI/AAI matrix tool (Rodriguez-R and Konstantinidis, 2016). Supplementary Figure S2 shows that the AAI values between S356^T^ and its closest strains range from 64.2 to 68.6%, evidencing that it belongs to a novel genus. The matrix also raises doubts about the classification of some species, such as *Polaribacter pacificus* or *Tenacibaculum agarivorans*, and those placed below in the table, but that observation is out of the scope of this study. Thus, we propose for strain S356^T^ a new genus and species with the name *Asprobacillus argos*.

A series of our isolates (F363^T^, F117^T^, F188^T^, F297^T^, F260^T^, and F225^T^) were placed together in the phylogenomic tree in a clearly differentiated node (Figure 3). Their closest neighbors are the type strains of *Salegentibacter mishustinae*, *S. agarivorans*, and *S. salarius*. We also included in the comparison *S. salegens*, the type species of the genus (McCammon and Bowman, 2000). The G + C content of these strains (38.3–40 mol%) is similar to that of *Salegentibacter* spp. (37.5–41 mol%), and all of them are catalase and oxidase-positive. While *S. mishustinae*, *S. agarivorans*, and *S. salarius* can grow at 4°C, only strains F188^T^ and F225^T^ can. Strain F363^T^, *S. agarivorans*, and *S. salarius* grow up to 40–41°C. Notably, strains F297^T^ and F260^T^ have a narrower temperature range compared to the rest. Regarding salinity, all strains grow at low salt percentages, but *Salegentibacter* species seem to tolerate higher salt concentrations (up to 18–20% w/v NaCl) than the novel isolates (up to 11% w/v sea salts). Assimilated carbon sources and enzymatic activities vary between these strains (Supplementary Table S8). Considering these data, the fact that none of our isolates have an AAI >75% with any *Salegentibacter* strain (Supplementary Table S5), and, particularly, accounting for the topology of the phylogenomic tree (Figure 3), we propose for strains F117^T^, F188^T^, F225^T^, F260^T^, F297^T^, and F363^T^ a new genus, *Autumnicola*, with six new species named: *A. musiva*, *A. patrickiae*, *A. psychrophila*, *A. lenta*, *A. edwardsiae*, and *A. tepida*, respectively. In addition to listing the main characteristics of the *Autumnicola* strains in Table 2 and Supplementary Table S8, we have made a drawing summarizing their roles in biogeochemical cycles (Supplementary Figure S3).

The next group of strains included F388^T^, P007^T^, and P059, which formed a node close to *Croceitalea dokdonensis* DOKDO 023^T^ (Figure 3). The type species of the genus *Croceitalea* is *C. eckloniae* (Lee et al., 2008), which we include in the comparison. The G + C content of strains P007^T^ and P059 is almost identical (34.7 and 34.6 mol%, respectively) and similar to strain F388^T^ (36.4 mol%), but in the case of *C. dokdonensis* and *C. eckloniae*, it is much higher: 59.4 and 66.5 mol%, respectively. Strains F388^T^ (28°C), P007^T^, and P059 (15–28°C) have a narrower temperature range than *C. dokdonensis* (12–38°C) and *C. eckloniae* (10–34°C). Salinity ranges are also narrow for these strains: none of them grows in less than 2% (w/v) sea salts nor in more than 5%. However, *C. dokdonensis* grows at 1–7% (w/v) sea salts, while *C. eckloniae* does at 0.5–7% (w/v) sea salts. While our isolates are oxidase-positive, *C. dokdonensis* and *C. eckloniae* are negative. Strains P007^T^ and P059 share most of the tested carbon sources and enzymatic activities, and they are fairly similar in these terms to F388^T^ but not to *C. dokdonensis* and *C. eckloniae* (Supplementary Table S8). Strains P007^T^ and P059 have an ANI of 98.95% (Supplementary Table S4), therefore they pertain to the same species (Jain et al., 2018). Given the topology of the phylogenomic tree (Figure 3) and the AAI values of almost 76% that the novel strains have with *C. dokdonensis* DOKDO 023^T^ (Supplementary Table S5), we propose two new species of the genus *Croceitalea*: *C. rosinachiae* for strain F388^T^ and *C. vernalis* for strain P007^T^.

The last strain of the *Flavobacteriaceae* phylogenomic tree is S334^T^, which clusters with *Pricia antarctica*, the only species of the genus *Pricia* (Yu et al., 2012). Strain S334^T^ has a higher G + C content (47 mol%) than *P. antarctica* (43.9 mol%). While strain S334^T^ thrives at 15–28°C, *P. antarctica* can survive at lower temperatures (0–25°C). Their salinity ranges are similar: 0.5–5% (w/v) sea salts for strain S334^T^ and 0.6–6% (w/v) NaCl for *P. antarctica*. Strain S334^T^ seems to assimilate many more carbon sources and have a broader set of enzymatic activities than *P. antarctica* (Supplementary Table S8). Taking into account these common and differential traits, considering the topology of the phylogenomic tree (Figure 3) and the AAI value of 80.87% (Supplementary Table S5) between S334^T^ and *P. antartica* DSM 2342^T^, we propose for strain S334^T^ a new species of *Pricia* with the name *Pricia mediterranea*.

### 4.4 Differential characteristics of the *Rhodothermia* strains

Strains F394^T^ and S365 appear close to *Rubrivirga marina* in the phylogenomic tree (Figure 4), which is the type species of the genus *Rubrivirga* (Park et al., 2013). We include in the comparison the only other species of that genus, *R. profundi* (Song et al., 2016), which was not included in the tree since the genome of its type strain is not available in public repositories. Strains F394^T^ and S365 have a higher G + C content (73.7 mol%) than *R. marina* (64.8–65.8 mol%) and *R. profundi* (66.2 mol%). Strains F394^T^ and S365 present PE and PG as their major polar lipids, but *R. marina* and *R. profundi* also include DPG. All of them display menaquinone 7 as their predominant respiratory quinone and do not contain flexirubin-type pigmentation. The temperature range of strains F394^T^ and S365 is 15–37°C, *R. marina* survives from 10°C to 37°C, and *R. profundi* has a broader range (4–42°C). While strain F394^T^ grows between 3 and 6% (w/v) sea salts, S365 does from 1.5 to 3% (w/v) sea salts, and *R. marina* and *R. profundi* develop between 1 and 5% (w/v) NaCl. All strains are oxidase and catalase positive except *R. marina*, which is catalase negative. Our strains have several differential results in API 20NE strip assimilation tests and present enzymatic profiles that diverge from *R. marina* and *R. profundi* (Supplementary Table S8). Interestingly, strains F394^T^ and S365 reduced nitrate to nitrite, but *R. profundi* did not show this capacity, and *R. marina* has not been reported to do so. F394^T^ and S365 have 98.88% ANI between them (Supplementary Table S4), and they are thus two strains of the same species (Jain et al., 2018). Taking into account all these data and the AAI values in Supplementary Table S5, we conclude that strain F394^T^ is a new species of *Rubrivirga*, with the proposed name *Rubrivirga mediterranea*.

### 4.5 Ecological relevance of the new strains

The strains characterized in this study contain several genes with important roles in carbon, nitrogen, sulfur, phosphorus, and hydrogen cycling in the oceans (Figure 5). The gene *coxL*, which encodes for the large subunit of CO dehydrogenase, is found in almost all the strains. The capacity of obtaining energy from CO through its oxidation is widespread and has been proposed as a relevant mechanism for long-term survival of heterotrophic bacteria in oligotrophic environments (Cordero et al., 2019). Strains P385^T^ and W335^T^, which have been characterized here as two members of the same novel genus, bear genes encoding for RuBisCO small and large subunits (genes *rbc*). They are, thus, potential important contributors to the carbon cycle via anaplerotic CO_2_ fixation (Newton et al., 2010). Interestingly, these two bacteria were isolated in winter and spring, and the *rbcL* gene was more abundant at the site of the study (Auladell et al., 2023). On the other hand, the *prd* gene (which encodes for proteorhodopsin) was found in strains P117^T^ (*Gammaproteobacteria*), F388^T^, P007^T^, P059 (three *Bacteroidia* grouping into the same genus), and S365 (*Rhodothermia*). Proteorhodopsin allows bacteria to obtain energy from light (Beja et al., 2000), and it is a relevant gene for long-term survival in low-nutrient environments such as the BBMO (Gómez-Consarnau et al., 2010). *Bacteroidia* and *Gammaproteobacteria* have been reported to contain this proton pump (Beja et al., 2000; Yoshizawa et al., 2014).

Furthermore, the gene *algL* could be detected in strains P117^T^, W364, W409^T^, W431^T^ (*Alteromonadaceae*), W332^T^, S356^T^ (*Flavobacteriaceae*), F388^T^, P007^T^, and P059 (closely related *Flavobacteriaceae*). This gene encodes for alginate lyase, which catalyzes the degradation of alginate (Wong et al., 2000). Alginate is a complex polysaccharide mainly found in the walls of brown algae and constitutes an important marine carbon source (Mabeau and Kloareg, 1987). Bacteria that degrade complex algal polysaccharides can make them available for fast-growing opportunistic bacteria to grow and potentially develop bacterial blooms (Teeling et al., 2012). Despite the genomic predictions, strains W409^T^, W431^T^, and W332^T^ were not seen to hydrolyze alginate experimentally, and W311^T^ hydrolyzed it weakly (Table 2), although it was not predicted to do so. In fact, detecting a gene in a genome does not necessarily correlate with its expression in the environment.

All the strains characterized here contain the *gdhA* gene encoding for glutamate dehydrogenase. This enzyme allows N assimilation through NH_4_ at the cost of α-ketoglutarate, and its expression has been seen to be enhanced when the N/C ratio is high (Hoch et al., 2006), acting as a balance between the carbon and nitrogen cycles. As commented above, although we found the *nifA* gene involved in the regulation of nitrogen fixation (Lei et al., 1999), we did not find among the novel strains the most relevant marker genes encoding for the different nitrogenases implicated in nitrogen fixation (*nifD*, *nifH*, or *nifK*). Several of the strains had potential for partial denitrification, but only three of them were seen to experimentally reduce nitrate to nitrite (Table 2): W431^T^, which contains periplasmic nitrate reductase genes *napA* and *napB*, F394^T^ and S365, for which no nitrate reduction genes were detected. All in all, several of the novel strains theoretically have the capacity to undergo partial denitrification, and we have confirmed the reduction of nitrate to nitrite in three of them, which makes them relevant members of the marine bacterial community in terms of the nitrogen cycle (Ferrera et al., 2015). Concerning other nitrogen compounds, although strains F158^T^ and W345^T^ contain the genes for the three subunits of urease, they gave negative results in API 20NE strips for this test (Table 2). In addition, strain W242^T^ lacked the *amt* gene encoding for a widespread ammonium transporter (Alonso-Sáez et al., 2020), and it is thus likely that these bacteria acquire N by other means that were not tested here.

The only gene related to the sulfur cycle that was found in these new isolates was *soxB*, a marker for the oxidation of sulfur compounds (Meyer et al., 2007), in the two strains pertaining to the novel genus *Spectribacter*. The *dmdA* gene, involved in dimethyl sulfoxide (DMSP) oxidation and widespread in marine bacteria (Moran et al., 2012), was not found in the novel strains. Moreover, the *pstS* gene, which is the marker for a phosphate transport system, was found mainly in *Alphaproteobacteria* and *Gammaproteobacteria*. Precisely these two groups have been reported to contain this gene, which seems to lack seasonality in the BBMO (Auladell et al., 2023) and has been proposed to be a general P uptake mechanism (Orchard et al., 2009). On the contrary, the *Flavobacteriaceae* strains exclusively contained the *pit* gene (except for F188^T^, which had both *pit* and *pstS*). The *pit* gene expression is enhanced under high P conditions, and it is related to copiotrophic *Gammaproteobacteria* and *Bacteroidia*, which would use it to cover their high energy demands (Alonso-Sáez et al., 2020). Among the novel strains, this gene was also found in some *Gammaproteobacteria* (precisely the ones affiliating with the copiotrophic family *Alteromonadaceae*) and two out of three *Alphaproteobacteria*. Interestingly, no phosphate acquisition genes were found in the *Rhodothermia* strains.

The only strain that seems to be capable of acquiring dissolved organic phosphorus (DOP) via a phosphonate transport system encoded by *phnE* is F158^T^, pertaining to the *Roseobacter* clade. Marine bacteria often rely on DOP to meet their phosphorus requirements, and *Rhodobacteraceae* have been characterized as important members of marine DOP cycling (Lockwood et al., 2022). While the most classical alkaline phosphatase *phoA* was found in 12 of the novel genomes, the marine extracellular phosphatases *phoD* and *phoX* (Luo et al., 2009; Sebastian and Ammerman, 2009) were found in 15 and 18 strains, respectively. Another protein used for DOP hydrolysis, the alkylphosphonate utilization protein encoded by *phnM*, was found only in strain F158^T^. *Rhodobacteraceae* was one of the groups reported to contain this gene in the BBMO (Auladell et al., 2023). In addition, the *ptxD* gene was present in all the strains except for six of the *Flavobacteriaceae*, indicating that phosphite could be a relevant P source for these bacteria (Martínez et al., 2012). Phospholipid remodeling is an important strategy in P-depleted environments such as the Mediterranean Sea, especially among *Flavobacteriaceae* and during the summer (Auladell et al., 2023). In concordance with these observations, the *plcP* gene was found in all strains except two of them (both *Alphaproteobacteria*). Finally, there was one strain, W335^T^, which contained the *hupL* gene. Hydrogen has recently drawn the attention of researchers as a notable energy source for marine bacteria, especially in environments with low primary production (Lappan et al., 2023).

The novel strains seem to be widespread in the global ocean (Figures 6, 7) and frequently constitute notorious proportions of the bacterial community, especially those belonging to the *Flavobacteriaceae*. Surprisingly, some of the strains were not detected in the Mediterranean Sea, where they were actually isolated. It could be that they had very low abundances during the Tara Oceans sampling, that took place in November 2009, quite separated from the sampling that yielded the novel isolates, occurring during the whole year in 2017.

### 4.6 Concluding remarks

Here, we have presented the full characterization of 23 novel bacterial species and six novel genera with potential relevant roles in biogeochemical cycling and widespread presence throughout the oceans. This study points to the need of revising the taxonomy of the genera *Glaciecola*, *Polaribacter*, and *Tenacibaculum*. In this study, the combination of bacterial cultures with sequencing-based approaches has proven to be a powerful tool to unveil new members of the marine microbial community with hypothetical ecological relevance. More studies combining cultures with sequencing are needed to describe the high number of uncharacterized bacterial species in the environment.

### 4.7 Description of *Tropicimonas omnivorans* sp. nov.

*Tropicimonas omnivorans* (om.ni.vo’rans. L. neut. adj. *omne*, everything; L. inf. v. *vorare*, to devour; N.L. part. adj. *omnivorans*, eating everything).

Cells are rod-shaped, short and thicker by the sides, and non-motile. Colonies on Marine Agar are small, opaque, pink-colored, and non-mucous; they grow between 15°C and 37°C and between 0.5 and 12% (w/v) sea salts; they do not grow in 2% (w/v) NaCl as the only salt; they are Gram-negative, oxidase-positive, and catalase-positive; they hydrolyze starch weakly and do not hydrolyze casein, alginate, DNA, or cellulose; and they do not grow in medium containing Tween-80. Among the carbon sources tested on simplified Basal Medium Agar grows on D-ribose, D-xylose, L-arabinose, D-glucose, D-mannose, D-trehalose, L-rhamnose, maltose, cellobiose, lactose, sucrose, melibiose, N-acetylglucosamine, glycerol, D-mannitol, D-glucuronate, acetate, pyruvate, citrate, 2-ketoglutarate, succinate, fumarate, malate, lactate, L-serine, L-alanine, L-glutamate, L-aspartate, and GABA. In API ZYM, it is positive for alkaline phosphatase, leucine and valine arylamidases, acid phosphatase, and naphtol AS-BI-phosphohydrolase. In API 20NE, it is positive for the assimilation of glucose, arabinose, mannose, mannitol, N-acetylglucosamine, maltose, gluconate, caprate, adipate, and malate. The predicted major polar lipids are DPG, PG, and PC. The predicted main respiratory quinone is ubiquinone 10.

The type strain is F158^T^ (= CECT 30792^T^ = CCM 9343^T^), which was isolated from microcosm experiments made with seawater from Blanes Bay. The DNA G + C content of the strain is 66.9 mol%, and its genome size is 3.94 Mbp. The GenBank accession numbers for the whole genome and 16S rRNA gene sequences are GCA_031846565 and OP342988, respectively.

### 4.8 Description of *Stakelama saccharophila* sp. nov.

*Stakelama saccharophila* (sac.cha.ro’phi.la. Gr. neut. n. *sakcharon*, sugar; N.L. fem. adj. suff. *-phila*, friend, loving; from Gr. fem. adj. *philê*, loving; N.L. fem. adj. *saccharophila*, sugar-loving).

Cells are thick, short rods often found in pairs. Motility is not observed. Colonies on Marine Agar are small, circular, convex, translucent, non-mucous, and pale yellow in color; they grow between 15°C and 37°C and between 0.5 and 4% (w/v) sea salts; they do not grow in 2% (w/v) NaCl as the only salt; they are Gram-negative, oxidase-positive, and catalase-positive; they hydrolyze Tween-80 and alginate (weakly), but not casein, starch, DNA, or cellulose. Among the carbon sources tested on simplified Basal Medium Agar grows on D-xylose, L-arabinose, D-glucose, D-fructose, D-mannose, D-trehalose, L-rhamnose, maltose, cellobiose, lactose, sucrose, melibiose, D-glucuronate, D-galacturonate, fumarate, malate, L-alanine, L-glutamate, and L-tyrosine. They grow weakly on acetate. In API 20NE, it is positive for aesculin hydrolysis and for the assimilation of glucose, arabinose, mannose, mannitol, N-acetylglucosamine, maltose, gluconate, adipate, malate, and phenylacetate. The predicted major polar lipids are DPG, PG, and PI. The predicted main respiratory quinone is ubiquinone 10.

The type strain is W311^T^ (= CECT 30796^T^ = CCM 9337^T^), which was isolated from microcosm experiments made with seawater from Blanes Bay. The DNA G + C content of the strain is 65.8 mol%, and its genome size is 3.19 Mbp. The GenBank accession numbers for the whole genome and 16S rRNA gene sequences are GCA_032229225 and OP344261, respectively.

### 4.9 Description of *Croceicoccus esteveae* sp. nov.

*Croceicoccus esteveae*, named after Isabel Esteve (1947–2020) in honor of her relevant career in microbiology teaching and research at the Universitat Autònoma de Barcelona (es.te’ve.ae. N.L. gen. fem. n. esteveae).

Cells are cocci or coccobacilli surrounded by fibrous material. Motility is not observed. Colonies on Marine Agar are small, circular, convex, translucent, non-mucous, and bright yellow in color; they grow between 15°C and 28°C and between 0.5 and 5% (w/v) sea salts; they do not grow in 2% (w/v) NaCl as the only salt; they are Gram-negative, oxidase-positive, and catalase-positive; they weakly hydrolyze starch and do not hydrolyze alginate, casein, DNA, or cellulose; they do not grow in Agar Tween-80; and they do not grow in any of the carbon sources tested on simplified Basal Medium Agar. In API 20NE, it is positive for the assimilation of glucose, arabinose, mannose, mannitol, N-acetylglucosamine, gluconate, caprate, adipate, malate, and phenylacetate. The predicted major polar lipids are PE, DPG, PG, and PI. The predicted main respiratory quinone is ubiquinone 10.

The type strain is F390^T^ (= CECT 30821^T^ = CCM 9349^T^), which was isolated from microcosm experiments made with seawater from Blanes Bay. The DNA G + C content of the strain is 60.7 mol%, and its genome size is 2.86 Mbp. The GenBank accession numbers for the whole genome and 16S rRNA gene sequences are GCA_031846435 and OP343189, respectively.

### 4.10 Description of *Banduia* gen. nov.

*Banduia* (Ban.du’ia. N.L. fem. n. *Banduia*, named after the Gallaecian and Lusitanian goddess of water Bandua).

Cells are Gram-negative bacilli; they are oxidase-positive and catalase-positive; they are mesophilic and slightly halophilic; they require sea salts for their growth; and they are aerobic and chemoorganotrophic. The predicted major polar lipids are DPG, PG, and PE. The predicted main respiratory quinone is ubiquinone 8. The DNA G + C content is 64 mol%.

Affiliated to family *Algiphilaceae* in class *Gammaproteobacteria*. The type species is *Banduia mediterranea*.

### 4.11 Description of *Banduia mediterranea* sp. nov.

*Banduia mediterranea* (me.di.ter.ra’ne.a. L. fem. adj. *mediterranea*, belonging to the Mediterranean Sea).

The description is as for the genus, with the following additions: cells are thick bacilli. Motility is not observed. Colonies on Marine Agar are small, circular, convex, transparent, and non-mucous, turning opaque, mucous, and white after 2 weeks; they grow between 15°C and 37°C and between 0.5 and 5% (w/v) sea salts, with weak growth in 6%; they do not grow in 2% (w/v) NaCl as the only salt; they are Gram-negative, oxidase-positive, and catalase-positive; they do not hydrolyze any of the tested compounds. Among the carbon sources tested on simplified Basal Medium Agar grows on maltose, N-acetylglucosamine, and butyrate; they grow weakly on acetate, 3-hydroxybutyrate, and L-citrulline. In API 20NE, it is positive for the assimilation of glucose, arabinose, mannose, mannitol, N-acetylglucosamine, maltose, gluconate, caprate, adipate, malate, and phenylacetate.

The type strain is W345^T^ (= CECT 30811^T^ = CCM 9345^T^), which was isolated from microcosm experiments made with Blanes Bay seawater. The DNA G + C content of the strain is 64 mol%, and its genome size is 3.84 Mbp. The GenBank accession numbers for the whole genome and 16S rRNA gene sequences are GCA_031846245 and OP344286, respectively.

### 4.12 Description of *Spectribacter* gen. nov.

*Spectribacter* (Spec.tri.bac’ter. L. neut. n. *spectrum*, as in specter, ghost; L. vowel *-i-*, connecting vowel; N.L. masc. n. *bacter*, rod or staff and, in biology, a bacterium; *Spectribacter*, a bacterium of a ghostly appearance, referring to the transparent, flat aspect of its colonies).

Cells are Gram-negative bacilli; they are oxidase-positive and catalase-positive; they are mesophilic and slightly halophilic; they require sea salts for their growth; they are aerobic and chemoorganotrophic. The predicted major polar lipids are PE, DPG, and PG. The predicted main respiratory quinone is ubiquinone 8. The DNA G + C content is 67.5–67.6 mol%.

They belong to the family *Salinispheraceae* in the class *Gammaproteobacteria*. The type species is *Spectribacter hydrogenooxidans*.

### 4.13 Description of *Spectribacter hydrogenooxidans* sp. nov.

*Spectribacter hydrogenooxidans* (hy.dro.ge.no.o’xi.dans. N.L. neut. n. *hydrogenum*, hydrogen; N.L. pres. part. *oxidans*, oxidizing; N.L. part. adj. *hydrogenooxidans*, hydrogen-oxidizing).

The description is as for the genus, with the following additions. Cells are curved bacilli sometimes forming small chains. Motility is not observed. Colonies on Marine Agar are small, circular, plain, non-mucous, and transparent that turn whiter with time; they grow between 15°C and 37°C and between 0.5 and 8% (w/v) sea salts, with weak growth in 9 and 10%; they do not grow in 2% (w/v) NaCl as the only salt; they are Gram-negative, oxidase-positive, and catalase-positive; they do not hydrolyze any of the tested compounds, and they do not grow in Tween-80 or DNA hydrolysis plates. Among the carbon sources tested on simplified Basal Medium Agar, grows on N-acetylglucosamine, acetate, pyruvate, malate, lactate, 3-hydroxybutyrate, L-arginine, L-glutamate, and L-ornitine. They grow weakly on D-trehalose, L-rhamnose, melibiose, glycerol, D-mannitol, D-sorbitol, D-gluconate, citrate, 2-ketoglutarate, succinate, fumarate, L-alanine, L-aspartate, L-citrulline, and GABA. In API 20NE, it is positive for the assimilation of arabinose, mannose, mannitol, N-acetylglucosamine, gluconate, adipate, malate, and phenylacetate. Its genome contains the *hupL* gene for hydrogen oxidation.

The type strain is W335^T^ (= CECT 30818^T^ = CCM 9350^T^), which was isolated from microcosm experiments with Blanes Bay seawater. The DNA G + C content of the strain is 65.6 mol%, and its genome size is 3.29 Mbp. The GenBank accession numbers for the whole genome and 16S rRNA gene sequences are GCA_031846275 and OP344279, respectively.

### 4.14 Description of *Spectribacter acetivorans* sp. nov.

*Spectribacter acetivorans* (a.ce.ti.vo’rans. L. neut. n. *acetum*, vinegar; L. pres. part. *vorans*, devouring; N.L. part. adj. *acetivorans*, acetate-consuming).

The description is as for the genus, with the following additions. Cells are curved bacilli, sometimes found in pairs. Motility is not observed. Colonies on Marine Agar are small, circular, plain, non-mucous, transparent-white in color; they grow between 15°C and 37°C and between 1.5 and 11% (w/v) sea salts, with weak growth in 1%; they do not grow in 2% (w/v) NaCl as the only salt; they are Gram-negative, oxidase-positive, and catalase-positive; they do not hydrolyze any of the tested compounds, and they do not grow in Tween-80 agar. Among the carbon sources tested on simplified Basal Medium Agar grows on saccharose, N-acetylglucosamine, acetate, pyruvate, 3-hydroxybutyrate, L-arginine, L-aspartate, L-glutamate, L-histidine, L-citrulline, and L-ornithine. They grow weakly on D-trehalose, L-rhamnose, maltose, lactose, melibiose, D-mannitol, D-sorbitol, D-gluconate, citrate, 2-ketoglutarate, succinate, fumarate, malate, lactate, L-alanine, and GABA. In API 20NE, it is positive for the assimilation of arabinose, mannose, mannitol, N-acetylglucosamine, gluconate, adipate, malate, and phenylacetate.

The type strain is P385^T^ (= CECT 30819^T^ = CCM 9351^T^), which was isolated from microcosm experiments with Blanes Bay seawater. The DNA G + C content of the strain is 65.7 mol%, and its genome size is 3.3 Mbp. The GenBank accession numbers for the whole genome and 16S rRNA gene sequences are GCA_031846305 and OP343522, respectively.

### 4.15 Description of *Glaciecola petra* sp. nov.

*Glaciecola petra* (pe’tra. L. gen. fem. n. *petra*, stone, referring to the aspect of its colonies).

Cells are rods that form occasional chains. Motility is not observed. Colonies on Marine Agar are medium-sized, spherical, opaque, hard, and cream-colored; they grow between 15°C (weakly) and 28°C and between 3 and 4% (w/v) sea salts; they do not grow in 2% (w/v) NaCl as the only salt; they are Gram-negative, oxidase-positive, and catalase-positive; they hydrolyze alginate, but not cellulose; they do not grow in medium with casein, starch, Tween-80, or DNA. Among the carbon sources tested on simplified Basal Medium Agar, it grows weakly on 2-ketoglutarate, succinate, fumarate, malate, 3-hydroxybutyrate, and L-citrulline. In API 20NE, it is positive for hydrolysis of aesculin, β-galactosidase activity, and the assimilation of glucose, arabinose, mannose, mannitol, N-acetylglucosamine, maltose, gluconate, caprate, adipate, malate, and phenylacetate. The predicted major polar lipids are PE and PG. The predicted main respiratory quinone is ubiquinone 8.

The type strain is P117^T^ (= CECT 30809^T^ = CCM 9332^T^), which was isolated from microcosm experiments with Blanes Bay seawater. The DNA G + C content of the strain is 39.9 mol%, and its genome size is 4.05 Mbp. The GenBank accession numbers for the whole genome and 16S rRNA gene sequences are GCA_031846345 and OP343310, respectively.

### 4.16 Description of *Brumicola* gen. nov.

*Brumicola* (Bru.mi’co.la. L. fem. n. *bruma*, winter; L. masc./fem. n. suff. *-cola*, an inhabitant; from L. masc./fem. n. *incola*, dweller; N.L. fem. n. *Brumicola*, a dweller of winter, after the season of isolation).

Cells are Gram-negative bacilli; they are oxidase-positive and catalase-positive; they are mesophilic and slightly halophilic; they require sea salts for their growth; they are aerobic and chemoorganotrophic. The predicted major polar lipids are PE and PG. The predicted main respiratory quinone is ubiquinone 8. The DNA G + C content is 39.8–44 mol%.

They belong to the family *Alteromonadaceae* in the class *Gammaproteobacteria*. The type species is *Brumicola pallidula*.

### 4.17 Description of *Brumicola pallidula* comb. nov.

*Brumicola pallidula* (pal.lid’u.la. L. fem. adj. *pallidula*, somewhat pale, referring to the weak pigmentation of the species when growing on plates).

Basonym: *Glaciecola pallidula* Bowman et al., 1998.

The description is as given for the basonym in Bowman et al. (1998).

The type strain is IC079^T^ (= ACAM 615^T^ = ATCC 700757^T^ = CIP 105819^T^ = DSM 14239^T^). The GenBank accession numbers for the whole genome and 16S rRNA gene sequences are GCA_000428905 and FR746107, respectively.

### 4.18 Description of *Brumicola nitratireducens* comb. nov.

*Brumicola nitratireducens* (ni.tra.ti.re.du’cens. N.L. masc. n. *nitras*, nitrate; L. pres. part. *reducens*, converting to a different state; N.L. part. adj. *nitratireducens*, reducing nitrate).

Basonym: *Glaciecola nitratireducens* Baik et al., 2006.

The description is as given for the basonym in Baik et al. (2006).

The type strain is FR1064^T^ (= JCM 12485^T^ = KCTC 12276^T^). The GenBank accession numbers for the whole genome and 16S rRNA gene sequences are GCA_000226565 and AY787042, respectively.

### 4.19 Description of *Brumicola blandensis* sp. nov.

*Brumicola blandensis* (blan.den’sis. N.L. masc./fem. adj. *blandensis*, pertaining to Blande or Blanda, the name the Romans used for the city of Blanes, which has given its name to the Bay of Blanes, where the type strain was isolated).

The description is as for the genus, with the following additions. Cells are rod-shaped. Motility is not observed. Colonies on Marine Agar are big, circular, convex, translucent, mucous, and cream-colored; they grow between 4°C and 37°C (one of the strains grows weakly at this temperature) and between 2 and 9% (w/v) sea salts; they do not grow in 2% (w/v) NaCl as the only salt; they are Gram-negative, oxidase-positive, and catalase-positive; they hydrolyze casein, starch, DNA, and alginate (strain W364), but not cellulose. Among the carbon sources tested on simplified Basal Medium Agar grows on acetate, pyruvate, butyrate, citrate, 2-ketoglutarate, succinate, fumarate, lactate, 3-hydroxybutyrate, L-arginine, L-serine, L-histidine, L-serine, and L-citrulline. Cellobiose, melibiose, and malate are assimilated by one of the two strains. It grows weakly on glycerol, D-mannitol, D-sorbitol, D-gluconate, L-aspartate, GABA, and L-threonine. Arabinose, lactose, sucrose, propionate, and L-glutamate are weakly assimilated by one of the two strains. In API ZYM, it is positive for alkaline phosphatase, leucine arylamidase, trypsin, α-chemotrypsin, acid phosphatase, and naphtol AS-BI phosphohydrolase. In API 20NE, it is positive for hydrolysis of aesculin and gelatin (strain W364), β-galactosidase activity, and the assimilation of glucose (varies among strains), arabinose, mannose, mannitol, N-acetylglucosamine, maltose, gluconate, caprate (varies among strains), adipate, malate, and phenylacetate.

The type strain is W409^T^ (= CECT 30798^T^ = CCM 9336^T^), which was isolated from microcosm experiments with Blanes Bay seawater. The DNA G + C content of the strain is 42.6 mol%, and its genome size is 4.01 Mbp. The GenBank accession numbers for the whole genome and 16S rRNA gene sequences are GCA_031846165 and OP344346, respectively. Strain W364 also belongs to this species.

### 4.20 Description of *Thalassotalea castellviae* sp. nov.

*Thalassotalea castellviae* (cas.tell’vi.ae, N.L. gen. fem. n. *castellviae*, in honor of Josefina Castellví, prominent biologist and oceanographer from the Institut de Ciències del Mar, CSIC).

Cells are rod-shaped with observed polar motility. Colonies on Marine Agar are big, circular, convex, translucent, mucous, and cream-colored; they grow between 4°C and 28°C with weak results at 37°C, and between 1.5 and 8% (w/v) sea salts; they do not grow in 2% (w/v) NaCl as the only salt; they are Gram-negative, oxidase-positive, and catalase positive; they hydrolyze casein and starch, but not alginate or cellulose; and they do not grow in medium with Tween-80 nor DNA. Among the carbon sources tested on simplified Basal Medium Agar grows on pyruvate and propionate, and weakly assimilated D-fructose. API 20NE, it is positive for reduction of nitrate to nitrite, hydrolysis of aesculin and gelatin, and assimilation of glucose, arabinose, mannose, mannitol, N-acetylglucosamine, maltose, gluconate, caprate, adipate, and malate.

The type strain is W431^T^ (= CECT 30799^T^ = CCM 9335^T^), which was isolated from microcosm experiments made with Blanes Bay seawater. The DNA G + C content of the strain is 37.2 mol%, and its genome size is 3.96 Mbp. The GenBank accession numbers for the whole genome and 16S rRNA gene sequences are GCA_031846185 and OP344367, respectively.

### 4.21 Description of *Patiriisocius hiemis* sp. nov.

*Patiriisocius hiemis* (hi’e.mis. L. gen. masc. n. *hiemis*, from the cold, from winter, related to the season of isolation).

Cells are thin rods that group in long chains. Motility is not observed. Colonies on Marine Agar are small, circular, convex, translucent, non-mucous, and intense yellow in color; they do not produce flexirubin-type pigments; they grow between 15°C and 37°C and between 3 and 8% (w/v) sea salts, with weak results in 2%; they do not grow in 2% (w/v) NaCl as the only salt; they are Gram-negative, oxidase-positive, and catalase positive; they hydrolyze casein and DNA, but not alginate, starch, or cellulose; and they do not grow in Agar Tween-80. Among the carbon sources tested on simplified Basal Medium Agar grows on L-arginine and grows weakly on acetate. In API 20NE, it is positive for hydrolysis of gelatin and assimilation of glucose, arabinose, mannose, mannitol, N-acetylglucosamine, maltose, gluconate, caprate, adipate, and malate. The predicted major polar lipid is PE. The predicted main respiratory quinone is menaquinone 6.

The type strain is W242^T^ (= CECT 30795^T^ = CCM 9333^T^), which was isolated from microcosm experiments made with Blanes Bay seawater. The DNA G + C content of the strain is 33.7 mol%, and its genome size is 2.84 Mbp. The GenBank accession numbers for the whole genome and 16S rRNA gene sequences are GCA_031846325 and OP344205, respectively.

### 4.22 Description of *Urechidicola vernalis* sp. nov.

*Urechidicola vernalis* (ver.na’lis. L. fem. adj. *vernalis*, belonging to the spring, the season when the type strain was isolated).

Cells are thin rods forming very long and sinuous chains. Motility is not observed. Colonies on Marine Agar are medium-sized, circular, convex, translucent, mucous, and yellow in color; they do not produce flexirubin-type pigments; they grow between 4°C and 28°C and between 3 and 4% (w/v) sea salts; they do not grow in 2% (w/v) NaCl as the only salt; they are Gram-negative, oxidase-positive, and catalase negative; they hydrolyze starch, but not casein, alginate, or cellulose; they do not grow in medium with Tween-80 or DNA; and they do not grow in any of the carbon sources tested on simplified Basal Medium Agar. In API 20NE, it is positive for assimilation of glucose, arabinose, mannose, mannitol, N-acetylglucosamine, maltose, gluconate, caprate, adipate, malate, and phenylacetate. The predicted major polar lipid is PE. The predicted main respiratory quinone is menaquinone 6.

The type strain is P050^T^ (= CECT 30802^T^ = CCM 9340^T^), which was isolated from microcosm experiments made with Blanes Bay seawater. The DNA G + C content of the strain is 33.5 mol%, and its genome size is 3.14 Mbp. The GenBank accession numbers for the whole genome and 16S rRNA gene sequences are GCA_031846385 and OP343250, respectively.

### 4.23 Description of *Microcosmobacter* gen. nov.

*Microcosmobacter* (Mi.cro.cos.mo.bac’ter. Gr. masc. adj. *mikros*, small; N.L. masc. n. Gr. masc. n. *kosmos*, universe or world; N.L. masc. n. *bacter*, a rod; N.L. masc. n. *Microcosmobacter*, a rod from a microcosm, in reference to the isolation site, a microcosm experiment).

Cells are Gram-negative bacilli; they are oxidase-positive and catalase-positive; they are mesophilic and slightly halophilic; they require sea salts for their growth; and they are aerobic and chemoorganotrophic. The predicted major polar lipid is PE. The predicted main respiratory quinone is menaquinone 6. They do not produce flexirubin-type pigments. The DNA G + C content is 32.5 mol%.

They belong to the family *Flavobacteriaceae* in the class *Bacteroidia*. The type species is *Microcosmobacter mediterraneus*.

### 4.24 Description of *Microcosmobacter mediterraneus* sp. nov.

*Microcosmobacter mediterraneus* (me.di.ter.ra’ne.us. L. masc. adj. *mediterraneus*, from the Mediterranean Sea).

The description is as for the genus, with the following additions. Cells are small, short rods. Motility is not observed. Colonies on Marine Agar are small, circular, convex, translucent, non-mucous, and yellow-orange in color; they do not produce flexirubin-type pigments; they grow at 28°C only and between 3 and 4% (w/v) sea salts; they do not grow in 2% (w/v) NaCl as the only salt; they are Gram-negative, oxidase-positive, and catalase positive; they hydrolyze casein, but not starch, alginate, or cellulose; do not grow in medium with Tween-80 or DNA; and they do not grow in any of the carbon sources tested on simplified Basal Medium Agar. In API 20NE, it is positive for assimilation of glucose, arabinose, mannose, mannitol, N-acetylglucosamine, maltose, gluconate, caprate, adipate, malate, and phenylacetate. The predicted major polar lipid is PE. The predicted main respiratory quinone is menaquinone 6.

The type strain is W332^T^ (= CECT 30810^T^ = CCM 9339^T^), which was isolated from microcosm experiments made with Blanes Bay seawater. The DNA G + C content of the strain is 32.5 mol%, and its genome size is 3 Mbp. The GenBank accession numbers for the whole genome and 16S rRNA gene sequences are GCA_031846265 and OP344277, respectively.

### 4.25 Description of *Asprobacillus* gen. nov.

*Asprobacillus* (As.pro.ba.ci’llus. Gr. adj. *Aspros*, white; L. masc. n. *bacillus*, small rod, N.L. masc. n. *Asprobacillus*, white rod).

Cells are Gram-negative bacilli; they are oxidase-positive and catalase-positive; they are mesophilic and slightly halophilic; they require sea salts for their growth; they are aerobic and chemoorganotrophic. The predicted major polar lipid is PE. The predicted main respiratory quinone is menaquinone 6. They do not produce flexirubin-type pigments. The DNA G + C content is 35.2 mol%.

They belong to the family *Flavobacteriaceae* in the class *Bacteroidia*. The type species is *Asprobacillus argos*.

### 4.26 Description of *Asprobacillus argos* sp. nov.

*Asprobacillus argos* (ar’gos. Gr. masc. adj. *argos*, slow, referring to its slow growth).

The description is as for the genus, with the following additions. Cells are thin rods. Motility is not observed. Colonies on Marine Agar are circular, convex, mucous, opaque, and white in color; they grow between 15°C and 28°C and between 1.5 and 3% (w/v) sea salts; they do not grow in 2% (w/v) NaCl as the only salt; they are Gram-negative, oxidase-positive, and catalase weakly positive; they hydrolyze starch, but not cellulose; they do not grow in medium with casein or Tween-80; they do not grow in any of the carbon sources tested on simplified Basal Medium Agar. In API ZYM, it is positive for alkaline phosphatase, leucine and valine arylamidases, acid phosphatase, and naphtol AS-BI phosphohydrolase.

The type strain is S356^T^ (= CECT 30474^T^ = CCM 9388^T^), which was isolated from microcosm experiments made in Blanes Bay seawater. The DNA G + C content of the strain is 35.2 mol%, and its genome size is 3.18 Mbp. The GenBank accession numbers for the whole genome and 16S rRNA gene sequences are GCA_032248395 and OP343904, respectively.

### 4.27 Description of *Autumnicola* gen. nov.

*Autumnicola* (Au.tum.ni’co.la. L. masc. n. *autumnus*, fall, autumn; L. masc./fem. n. suff. *-cola*, an inhabitant; from L. masc./fem. n. *incola*, dweller; N.L. fem. n. *Autumnicola*, autumn dweller, in reference of the season when the strains of this genus were isolated).

Cells are Gram-negative bacilli; they are oxidase-positive and catalase-positive; they are mesophilic and slightly halophilic; they require sea salts for their growth; they are aerobic and chemoorganotrophic. The predicted major polar lipid is PE. The predicted main respiratory quinone is menaquinone 6. They do not produce flexirubin-type pigments. The DNA G + C content is 38.3–40 mol%.

They belong to the family *Flavobacteriaceae* in the class *Bacteroidia*. The type species is *Autumnicola musiva*.

### 4.28 Description of *Autumnicola musiva* sp. nov.

*Autumnicola musiva* (mu’si.va, L. fem. adj. *musiva*, mosaic, related to the aspect of the cells observed through an optical microscope).

The description is as for the genus, with the following additions. Cells are short bacilli, often forming sinuous chains. Motility is not observed. Colonies on Marine Agar are small, circular, convex, translucid, mucous, and yellowish in color; they grow between 15°C and 37°C and between 0.5 and 7% (w/v) sea salts, with weak growth in 8 and 9%; they do not grow in 2% (w/v) NaCl as the only salt; they are Gram-negative, oxidase-positive, and catalase-positive; they hydrolyze DNA and weakly hydrolyze casein and starch; they do not hydrolyze alginate or cellulose; and they do not grow on medium with Tween-80. Among the carbon sources tested on simplified Basal Medium Agar grows on D-trehalose, L-rhamnose, cellobiose, lactose, sucrose, N-acetylglucosamine, and L-glutamate. In API 20NE, it is positive for hydrolysis of aesculin and assimilation of glucose, arabinose, mannose, mannitol, N-acetylglucosamine, maltose, gluconate, caprate, adipate, malate, citrate, and phenylacetate.

The type strain is F117^T^ (= CECT 30804^T^ = CCM 9344^T^), which was isolated from microcosm experiments made with Blanes Bay seawater. The DNA G + C content of the strain is 38.3 mol%, and its genome size is 4.52 Mbp. The GenBank accession numbers for the whole genome and 16S rRNA gene sequences are GCA_031846585 and OP342948, respectively.

### 4.29 Description of *Autumnicola tepida* sp. nov.

*Autumnicola tepida* (te’pi.da, L. fem. adj. *tepida*, warm).

The description is as for the genus, with the following additions. Cells are thin rods, frequently forming sinuous chains. Motility is not observed. Colonies on Marine Agar are small, circular, convex, translucent, non-mucous, orange in the center, and yellow in the margins; they grow between 15°C and 40°C and between 0.5 and 4% (w/v) sea salts; they do not grow in 2% (w/v) NaCl as the only salt; they are Gram-negative, oxidase-positive, and catalase-positive; they hydrolyze casein, Tween-80, and DNA and weakly hydrolyze starch; and they do not hydrolyze alginate or cellulose. Among the carbon sources tested on simplified Basal Medium Agar grows on D-trehalose, maltose, cellobiose, lactose, sucrose, melibiose, N-acetylglucosamine, and D-gluconate. In API 20NE, it is positive for hydrolysis of aesculin and gelatin, β-galactosidase activity, and assimilation of glucose, arabinose, mannose, mannitol, N-acetylglucosamine, maltose, adipate, malate, and phenylacetate.

The type strain is F363^T^ (= CECT 30820^T^ = CCM 9348^T^), which was isolated from microcosm experiments made with Blanes Bay seawater. The DNA G + C content of the strain is 40 mol%, and its genome size is 4.37 Mbp. The GenBank accession numbers for the whole genome and 16S rRNA gene sequences are GCA_031846485 and OP343172, respectively.

### 4.30 Description of *Autumnicola patrickiae* sp. nov.

*Autumnicola patrickiae* (pa.trick’i.ae, N.L. gen. fem. n. *patrickiae*, in honor of Ruth Myrtle Patrick (1907–2013), a botanist and limnologist specializing in diatoms and freshwater ecology).

The description is as for the genus, with the following additions. Cells are long rods, sometimes forming pairs or sinuous chains. Motility is not observed. Colonies on Marine Agar are small, circular, convex, translucent, non-mucous, and yellow in color; they grow between 4°C and 37°C and between 1.5 and 11% (w/v) sea salts, with weak growth in 1%; they do not grow in 2% (w/v) NaCl as the only salt; they are Gram-negative, oxidase-positive, and catalase positive; they hydrolyze DNA and weakly hydrolyze starch; they do not hydrolyze casein, alginate, or cellulose; and they do not grow in medium with Tween-80. Among the carbon sources tested on simplified Basal Medium Agar only grows on D-trehalose. In API 20NE, it is positive for hydrolysis of aesculin, β-galactosidase activity, and assimilation of glucose, arabinose, mannose, mannitol, N-acetylglucosamine, maltose, gluconate, caprate, adipate, malate, and phenylacetate.

The type strain is F188^T^ (= CECT 30805^T^ = CCM 9341^T^), which was isolated from microcosm experiments made with Blanes Bay seawater. The DNA G + C content of the strain is 38.4 mol%, and its genome size is 4.35 Mbp. The GenBank accession numbers for the whole genome and 16S rRNA gene sequences are GCA_031846525 and OP343012, respectively.

### 4.31 Description of *Autumnicola edwardsiae* sp. nov.

*Autumnicola edwardsiae* (ed.ward’si.ae, N.L. gen. fem. n. *edwardsiae*, in honor of Katrina Jane Edwards (1968–2014), pioneering geomicrobiologist also called ‘Mistress of the Dark World’ known for her studies on the organisms living below the ocean floor).

The description is as for the genus, with the following additions. Cells are short, thick rods, often found in small or long sinuous chains. Motility is not observed. Colonies on Marine Agar are small, circular, convex, translucent, non-mucous, and yellow in color; they grow between 15°C and 28°C and between 1 and 11% (w/v) sea salts, with weak growth in 12%; they do not grow in 2% (w/v) NaCl as the only salt; they Gram-negative, oxidase-positive, and catalase-positive; they hydrolyze DNA and weakly hydrolyze starch; they do not hydrolyze casein, alginate, or cellulose; and they do not grow in medium with Tween-80. Among the carbon sources tested on simplified Basal Medium Agar only grows weakly on lactose. In API ZYM, it is positive for alkaline phosphatase, leucine and valine arylamidases, acid phosphatase, and naphtol AS-BI phosphohydrolase. In API 20NE, it is positive for hydrolysis of aesculin and assimilation of glucose, arabinose, mannose, mannitol, N-acetylglucosamine, maltose, caprate, adipate, malate, and phenylacetate.

The type strain is F297^T^ (= CECT 30794^T^ = CCM 9342^T^), which was isolated from microcosm experiments made with Blanes Bay seawater. The DNA G + C content of the strain is 38.9 mol%, and its genome size is 3.83 Mbp. The GenBank accession numbers for the whole genome and 16S rRNA gene sequences are GCA_031846505 and OP343113, respectively.

### 4.32 Description of *Autumnicola lenta* sp. nov.

*Autumnicola lenta* (len.ta, L. fem. adj. *lenta*, referring to its slow growth).

The description is as for the genus, with the following additions. Cells are short, thick rods, sometimes thicker on the margins, often forming pairs or chains. Motility is not observed. Colonies on Marine Agar are small, circular, convex, translucent, non-mucous, and yellowish in color; they grow between 15°C and 28°C, with weak growth at 4°C, and between 1.5 and 11% (w/v) sea salts; they do not grow in 2% (w/v) NaCl as the only salt; they are Gram-negative, oxidase-positive, and catalase-positive; they hydrolyze DNA and weakly hydrolyze starch; they do not hydrolyze casein, alginate, or cellulose and did not grow in medium with Tween-80; and they do not grow on any of the carbon sources tested on simplified Basal Medium Agar. In API 20NE, it is positive for hydrolysis of aesculin and assimilation of glucose, arabinose, mannose, mannitol, N-acetylglucosamine, maltose, gluconate, adipate, malate, citrate, and phenylacetate.

The type strain is F260^T^ (= CECT 30806^T^ = CCM 9346^T^), which was isolated from microcosm experiments made with Blanes Bay seawater. The DNA G + C content of the strain is 38.6 mol%, and its genome size is 3.93 Mbp. The GenBank accession numbers for the whole genome and 16S rRNA gene sequences are GCA_031846515 and OP343076, respectively.

### 4.33 Description of *Autumnicola psychrophila* sp. nov.

*Autumnicola psychrophila* (psy.chro’phi.la. Gr. masc. adj. *psychros*, cold; Gr. masc. adj. *philos*, liking, loving; N.L. fem. adj. *psychrophila*, cold-liking, as it grows in low temperatures).

The description is as for the genus, with the following additions. Cells are long rods, often found in pairs. Motility is not observed. Colonies on Marine Agar are small, circular, convex, translucent, non-mucous, and yellow in color; they grow between 4°C and 37°C and between 0.5 and 11% (w/v) sea salts; they do not grow in 2% (w/v) NaCl as the only salt; they are Gram-negative, oxidase-positive, and catalase positive; they hydrolyze DNA and weakly hydrolyze starch; they do not hydrolyze casein, alginate, or cellulose; and they do not grow in medium with Tween-80. Among the carbon sources tested on simplified Basal Medium Agar grows on D-trehalose, maltose, and lactose. In API 20NE, it is positive for hydrolysis of aesculin, β-galactosidase activity, and assimilation of glucose, arabinose, mannose, mannitol, N-acetylglucosamine, maltose, gluconate, adipate, malate, and phenylacetate.

The type strain is F225^T^ (= CECT 30793^T^ = CCM 9347^T^), which was isolated from microcosm experiments made with Blanes Bay seawater. The DNA G + C content of the strain is 38.4 mol%, and its genome size is 3.96 Mbp. The GenBank accession numbers for the whole genome and 16S rRNA gene sequences are GCA_031846645 and OP343046, respectively.

### 4.34 Description of *Croceitalea rosinachiae* sp. nov.

*Croceitalea rosinachiae* (ro.si.nach’i.ae, N.L. fem. n. *rosinachiae*, in honor of Zoe Rosinach Pedrol (1894–1973), the first Spanish woman to get a Ph.D. in pharmacy and a pioneering feminist activist).

Cells are very thin rods that often form short chains. Motility is not observed. Colonies on Marine Agar are small, circular, convex, translucent, non-mucous, and intense orange in color; they do not produce flexirubin-type pigments; they grow at 28°C only and between 2 and 5% (w/v) sea salts; they do not grow in 2% (w/v) NaCl as the only salt; they are Gram-negative, oxidase-positive, and catalase-positive; they hydrolyze alginate, but not cellulose; and they do not grow in mediums with casein, starch, Tween-80, or DNA. Among the carbon sources tested on simplified Basal Medium Agar grows only on lactose. In API ZYM, it is positive for alkaline phosphatase, leucine and valine arylamidases, trypsin, acid phosphatase, naphtol AS-BI phosphohydrolase, α-glucosidase, and N-acetyl-β-glucosaminidase. In API 20NE, it is positive for hydrolysis of aesculin, β-galactosidase activity, and assimilation of mannose, mannitol, N-acetylglucosamine, maltose, gluconate, caprate, adipate, malate, and phenylacetate. The predicted major polar lipid is PE. The predicted main respiratory quinone is menaquinone 6.

The type strain is F388^T^ (= CECT 30807^T^ = CCM 9338^T^), which was isolated from microcosm experiments made with Blanes Bay seawater. The DNA G + C content of the strain is 36.4 mol%, and its genome size is 3.6 Mbp. The GenBank accession numbers for the whole genome and 16S rRNA gene sequences are GCA_031846445 and OP343188, respectively.

### 4.35 Description of *Croceitalea vernalis* sp. nov.

*Croceitalea vernalis* (ver.na’lis. L. fem. adj. *vernalis*, belonging to the spring, the season when the type strain was isolated).

Cells are very thin rods, often forming short to long chains. Motility is not observed. Colonies on Marine Agar are small, circular, convex, translucent, non-mucous, and intense orange in color; they do not produce flexirubin-type pigments; they grow between 15°C and 28°C and between 3 and 5% (w/v) sea salts. One of the strains has weak growth in 2 and 5% (w/v) sea salts. They do not grow in 2% (w/v) NaCl as the only salt; they are Gram-negative, oxidase-positive, and catalase-positive; they hydrolyze alginate but not starch, casein, or cellulose; and they do not grow in medium containing Tween-80 or DNA. Among the carbon sources tested on simplified Basal Medium Agar grows weakly on 2-ketoglutarate. In API ZYM, it is positive for alkaline phosphatase, esterase C4 (strain P007^T^, weakly), leucine and valine arylamidases, trypsin, acid phosphatase, naphtol AS-BI phosphohydrolase, α-glucosidase (strain P007^T^), and N-acetyl-β-glucosaminidase. In API 20NE, it is positive for hydrolysis of aesculin and assimilation of glucose, arabinose, mannose, mannitol, N-acetylglucosamine, maltose, gluconate, adipate, malate, and phenylacetate. The predicted major polar lipid is PE. The predicted main respiratory quinone is menaquinone 6.

The type strain is P007^T^ (= CECT 30801^T^ = CCM 9330^T^), which was isolated from microcosm experiments made with Blanes Bay seawater. The DNA G + C content of the strain is 34.7 mol%, and its genome size is 3.38 Mbp. The GenBank accession numbers for the whole genome and 16S rRNA gene sequences are GCA_031846405 and OP343207, respectively. Strain P059 also belongs to this species.

### 4.36 Description of *Pricia mediterranea* sp. nov.

*Pricia mediterranea* (me.di.ter.ra’ne.a. L. fem. adj. *mediterranea*, belonging to the Mediterranean Sea).

Cells are thin rods. Motility is not observed. Colonies on Marine Agar are circular, convex, with undulate margin, translucent, mucous, and intense orange in color; they do not produce flexirubin-type pigments; they grow between 15°C and 28°C and between 0.5 and 5% (w/v) sea salts; they do not grow in 2% (w/v) NaCl as the only salt; they are Gram-negative, oxidase-positive, and catalase weakly positive; they hydrolyze casein and Tween-80, but not starch or cellulose. Among the carbon sources tested on simplified Basal Medium Agar grows weakly on D-mannose, D-trehalose, L-rhamnose, maltose, lactose, mannitol, D-gluconate, salicilin, amigdalin, fumarate, 3-hydroxybutyrate, L-arginine, and L-ornithine. In API ZYM, it is positive for alkaline phosphatase, leucine, valine and cystine arylamidases, trypsin, acid phosphatase, naphtol AS-BI phosphohydrolase, β-galactosidase, α-glucosidase, β-glucosidase, N-acetyl-β-glucosaminidase, and α-mannosidase. In API 20NE, it is positive for hydrolysis of aesculin and β-galactosidase activity. The predicted major polar lipids are PE and DPG. The predicted main respiratory quinone is menaquinone 6.

The type strain is S334^T^ (= CECT 30471^T^ = CCM 9387^T^), which was isolated from microcosm experiments made with Blanes Bay seawater. The DNA G + C content of the strain is 47 mol%, and its genome size is 4.57 Mbp. The GenBank accession numbers for the whole genome and 16S rRNA gene sequences are GCA_032248455 and OP343883, respectively.

### 4.37 Description of *Rubrivirga litoralis* sp. nov.

*Rubrivirga litoralis* (li.to.ra’lis. L. masc./fem. adj. *litoralis*, coastal).

Cells are peanut-shaped rods with an important extracellular matrix. Big spheres are also observed on scanning electron microscopy. Motility is not observed. Colonies on Marine Agar are small, circular, convex, opaque, aqueous, and pale red in color that turns more intense with time; they grow between 15°C and 37°C. One of the strains grows between 3 and 6% (w/v) sea salts and the other does between 1.5 and 3% (w/v) sea salts, with weak growth at 4%; they do not grow in 2% (w/v) NaCl as the only salt; they are Gram-negative, oxidase-positive, and catalase-positive; they hydrolyze starch, but not alginate or cellulose; they do not grow in medium containing casein, Tween-80, or DNA; and they do not grow on any of the carbon sources tested on simplified Basal Medium Agar. In API ZYM, it is positive for alkaline phosphatase, leucine and valine arylamidases, α-chymotrypsin, acid phosphatase, naphtol AS-BI phosphohydrolase, and α-glucosidase. In API 20NE, it is positive for reduction of nitrate to nitrite and aesculin hydrolysis, β-galactosidase activity (strain S365), glucose, arabinose, mannose, mannitol, N-acetylglucosamine, maltose, gluconate, adipate, and phenylacetate (all the assimilations were positive in strain F394^T^). The predicted major polar lipids are PE and PG. The predicted main respiratory quinone is menaquinone 7.

The type strain is F394^T^ (= CECT 30808^T^ = CCM 9352^T^), which was isolated from microcosm experiments made with Blanes Bay seawater. The DNA G + C content of the strain is 73.7 mol%, and its genome size is 3.72 Mbp. The GenBank accession numbers for the whole genome and 16S rRNA gene sequences are GCA_031846415 and OP343191, respectively. Strain S365 also belongs to this species.

## Data availability statement

The 16S rRNA gene Sanger sequences can be found in GenBank under accessions OP342948, OP342988, OP343012, OP343046, OP343076, OP343113, OP343172, OP343188, OP343189, OP343191, OP343207, OP343250, OP343258, OP343310, OP343522, OP343883, OP343904, OP343913, OP344205, OP344261, OP344277, OP344279, OP344286, OP344303, OP344346, and OP344367. The genome sequences used in this study can be found in GenBank under BioProject PRJNA1016293. The Tara Oceans metagenomic datasets can be found in ENA under BioProject PRJEB1787 and PJREB9740.

## Author contributions

XR-V: Conceptualization, Data curation, Formal analysis, Investigation, Methodology, Writing – original draft, Writing – review & editing. TL: Data curation, Validation, Writing – review & editing. AB: Data curation, Investigation, Validation, Writing – review & editing. JG: Funding acquisition, Resources, Supervision, Validation, Writing – review & editing. OS: Funding acquisition, Resources, Supervision, Validation, Writing – review & editing. DA: Conceptualization, Data curation, Funding acquisition, Investigation, Methodology, Resources, Supervision, Validation, Writing – review & editing. MP: Conceptualization, Funding acquisition, Investigation, Methodology, Resources, Supervision, Validation, Writing – review & editing.

## Footnotes

^1^ http://www.bioinformatics.babraham.ac.uk/projects/fastqc

^2^ https://github.com/erikrikarddaniel/biomakefiles

^3^ https://github.com/ncbi/datasets

^4^ https://github.com/wwood/CoverM

## Supplementary material

SUPPLEMENTARY TABLE S1 Biogeochemically relevant genes screened in this study and their references.

SUPPLEMENTARY TABLE S2 Extended genomic characteristics of the strains, including information of the 16S rRNA gene sequences and the closest neighbors according to ANI (Average Nucleotide Identity), AAI (Average Aminoacid Identity) and dDDH (digital DNA-DNA hybridization) values.

SUPPLEMENTARY TABLE S3 Taxonomic classification of the novel strains from their 16S rRNA gene sequences and their genomes.

SUPPLEMENTARY TABLE S4 ANI (Average Nucleotide Identity) and AAI (Average Aminoacid Identity) values between the novel strains.

SUPPLEMENTARY TABLE S5 Full list of and digital DNA-DNA hybridization (dDDH), AAI (Average Aminoacid Identity) and ANI (Average Nucleotide Identity) values between the novel strains and their close relatives. The TYGS (Type Strain Genome Server) section includes all the genomes that were used to compute the phylogenomic trees of each group and their accession numbers.

SUPPLEMENTARY TABLE S6 Results for sole carbon source utilization in simplified Basal Medium Agar. w, weak growth; NT, not tested.

SUPPLEMENTARY TABLE S7 Taxogenomic characteristics (major polar lipids, respiratory quinones and gliding motility genes) of the strains as predicted from their genomes. MK, menaquinone; Q, ubiquinone; PE, phosphatidilethanolamine; PG, phosphatidilglycerol; DPG, diphosphatidilglyerol; PI, phosphatidil m-inositol; PC, phosphatidilcholine.

SUPPLEMENTARY TABLE S8 Comparison of phenotypic and genomic characteristics of the strains and their closest relatives. Positive results are colored in red, weakly positive results are colored in pale red and negative results are colored in blue. NT, not tested (or not reported in the literature); NA, non-applicable (mostly if the given test was not performed in the strain of this study); NG, no growth (for hydrolysis tests); w, weak results. MK, menaquinone; Q, ubiquinone; PE, phosphatidilethanolamine; PG, phosphatidilglycerol; DPG, diphosphatidilglyerol; PI, phosphatidil m-inositol; PC, phosphatidilcholine.

SUPPLEMENTARY FIGURE S1 AAI (Average Aminoacid Identity) matrix of strains P117^T^, W364, W409^T^ and their closest neighbors obtained with the Kostas Lab AAI/ANI matrix tool (Rodriguez-R and Konstantinidis, 2016).

SUPPLEMENTARY FIGURE S2 AAI (Average Aminoacid Identity) matrix of strain S365^T^ and its closest neighbors obtained with the Kostas Lab AAI/ANI matrix tool (Rodriguez-R and Konstantinidis, 2016).

SUPPLEMENTARY FIGURE S3 Graphical representation of the relevance of the novel genus *Autumnicola* in the cycles of carbon, nitrogen and phosphorus. The genes that appear in the drawing are contained by at least one of the members of this genus.
