# Supplementary figures and images for: Genomic and phenotypic characterization of 26 novel marine bacterial strains with relevant biogeochemical roles and widespread presence across the global ocean
Source: Front Microbiol. 2024 May 28;15:1407904. doi: 10.3389/fmicb.2024.1407904 (PMC11165706; doi:10.3389/fmicb.2024.1407904)

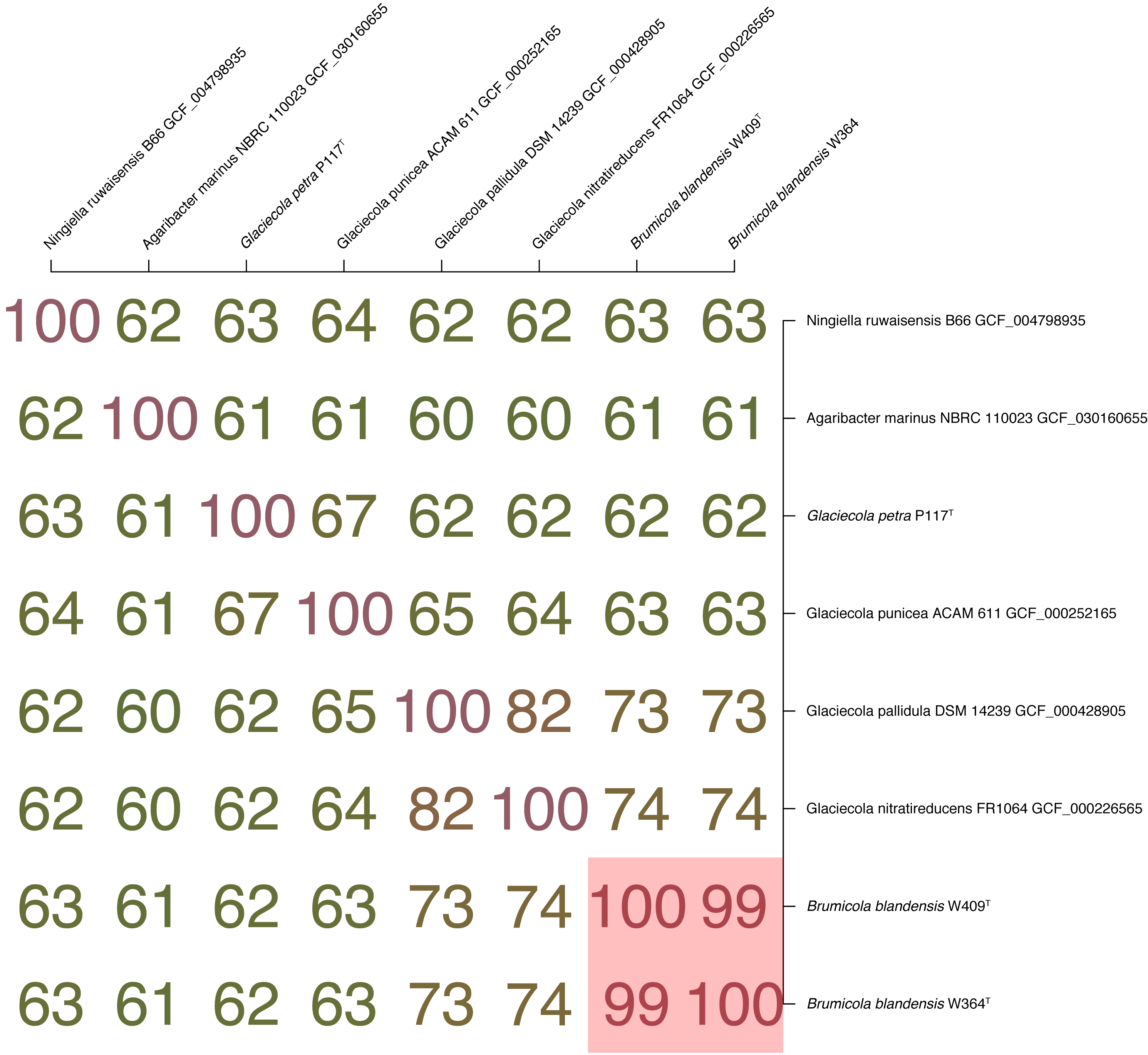

Supplement: SUPPLEMENTARY FIGURE S1 — AAI (Average Aminoacid Identity) matrix of strains P117T, W364, W409T and their closest neighbors obtained with the Kostas Lab AAI/ANI matrix tool (Rodriguez-R and Konstantinidis, 2016). [file Image_1.PNG]

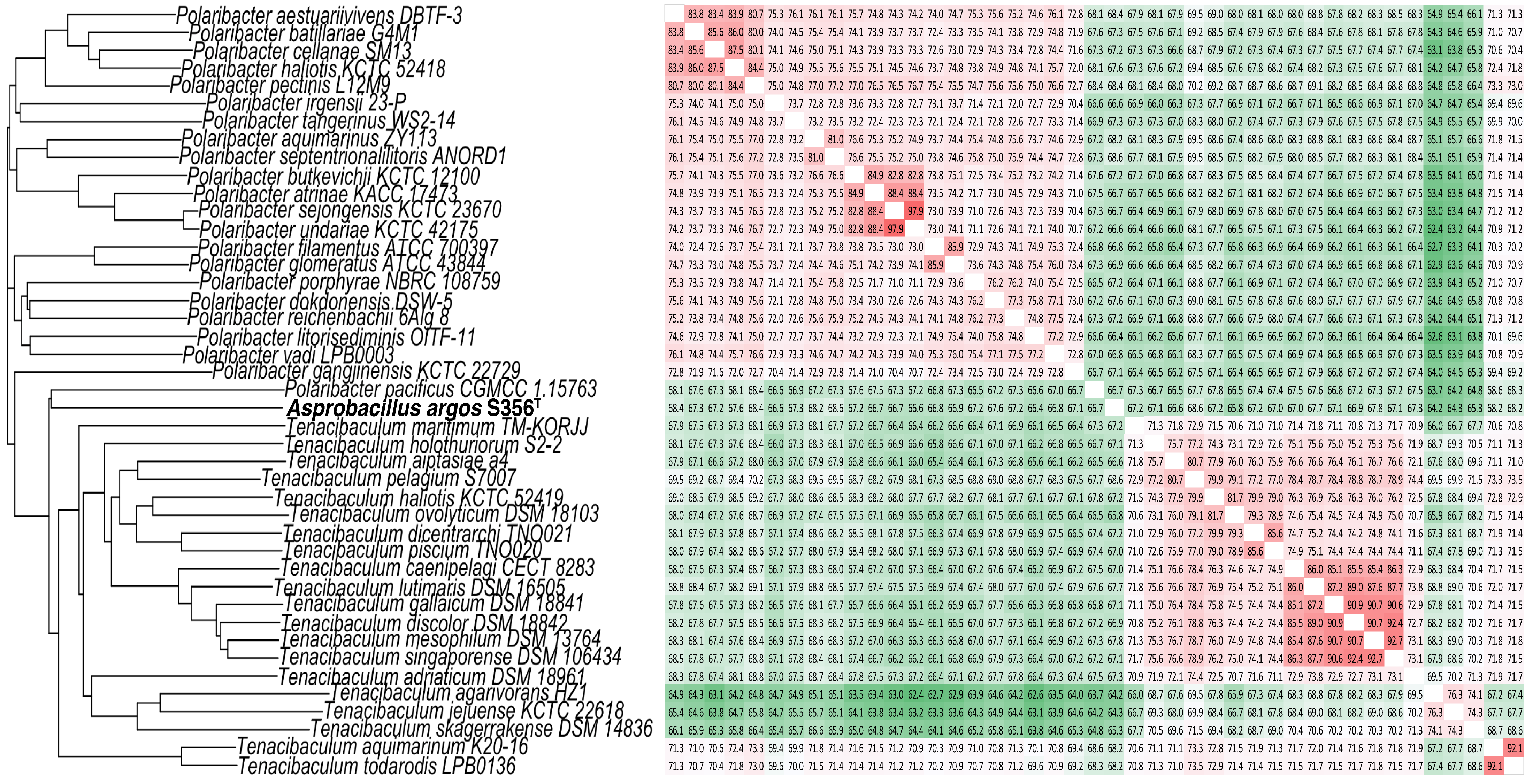

Supplement: SUPPLEMENTARY FIGURE S2 — AAI (Average Aminoacid Identity) matrix of strain S365T and its closest neighbors obtained with the Kostas Lab AAI/ANI matrix tool (Rodriguez-R and Konstantinidis, 2016). [file Image_2.PNG]

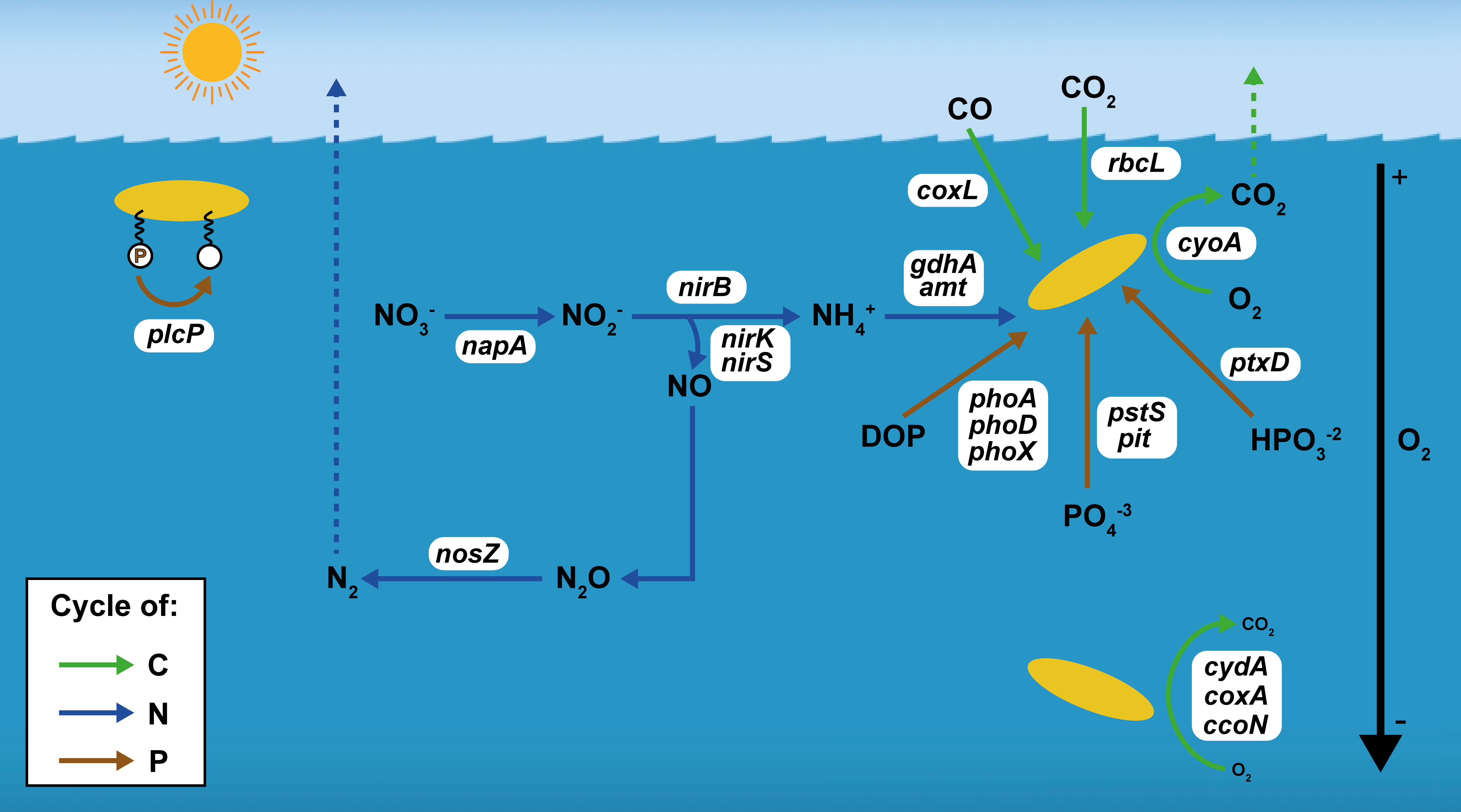

Supplement: SUPPLEMENTARY FIGURE S3 — Graphical representation of the relevance of the novel genus Autumnicola in the cycles of carbon, nitrogen and phosphorus. The genes that appear in the drawing are contained by at least one of the members of this genus. [file Image_3.TIF]
